# A Meta-Analysis of Rupture Risk for Intracranial Aneurysms 10 mm or Less in Size Selected for Conservative Management Without Repair

**DOI:** 10.3389/fneur.2021.743023

**Published:** 2022-02-17

**Authors:** Ronil V. Chandra, Julian Maingard, Lee-Anne Slater, Nicholas K. Cheung, Leon T. Lai, Seana L. Gall, Amanda G. Thrift, Thanh G. Phan

**Affiliations:** ^1^NeuroInterventional Radiology, Monash Medical Centre, Monash Health, Melbourne, VIC, Australia; ^2^School of Clinical Sciences at Monash Health, Monash University, Melbourne, VIC, Australia; ^3^Department of Neurosurgery, Monash Medical Centre, Monash Health, Melbourne, VIC, Australia; ^4^Menzies Institute for Medical Research, University of Tasmania, Hobart, TAS, Australia; ^5^Stroke and Ageing Research, Department of Medicine, School of Clinical Sciences at Monash Health, Monash University, Melbourne, VIC, Australia; ^6^Department of Neurology, Monash Medical Centre, Monash Health, Melbourne, VIC, Australia

**Keywords:** evidence based medicine (EBM), Systematic Reviews and Meta-Analyses, subarachnoid hemorrhage, cerebral aneurysm, intracranial aneurysm

## Abstract

**Background:**

Small unruptured intracranial aneurysms (UIAs) are considered to have low risk of rupture. The proportion of UIAs measuring 10 mm or less in size that rupture when selected for conservative management without repair is not well known. The aim of this study is to determine the proportion of UIAs that rupture by size threshold from ≤10 to ≤3 mm when selected for management without repair and to determine the level of precision and sources of heterogeneity in the rupture risk estimate.

**Methods:**

This study was prospectively registered with the International Prospective Register of Systematic Reviews (PROSPERO) (CRD42019121522). The Ovid MEDLINE, EMBASE, Web of Science Core Collection, and the Cochrane Central Register of Controlled Trials were searched (inception to August 2020). Studies with longitudinal follow-up of patients with UIAs ( ≤10 mm to ≤3 mm) without endovascular or neurosurgical repair were eligible. We included studies, which provided details of aneurysm size and in which UIA rupture was reported as an outcome. The primary outcome of the pooled proportion of UIA rupture during follow-up was synthesized with random-effects meta-analysis; heterogeneity was explored using meta-regression.

**Results:**

A total of 31 studies that included 13,800 UIAs ≤10 mm in size were eligible for data synthesis. The pooled proportion of ≤10 mm UIAs that ruptured when managed without repair was 1.1% (95% CI 0.8–1.5; *I*^2^ = 52.9%) over 3.7 years. Findings were consistent in sensitivity analyses at all the size stratified thresholds including ≤5 and ≤3 mm; rupture occurred in 1.0% (95% CI 0.8–1.3; *I*^2^ = 0%) of 7,280 ≤5 mm UIAs and 0.8% (95% CI 0.4–1.5; *I*^2^ = 0%) of 1,228 ≤3 mm UIAs managed without repair. In higher quality studies with lower risk of bias, rupture occurred in 1.8% (95% CI 1.5–2.0; *I*^2^ = 0%) over 3.9 years. In meta-regression, aneurysm size, shape, anatomical location, and exposure to prior subarachnoid hemorrhage were not identified as sources of heterogeneity.

**Conclusion:**

For every 1,000 UIAs that are 10 mm or less in size and selected for conservative management without repair, between 8 and 15 UIAs are estimated to rupture over 3.7 years. When stratified by size, these pooled rupture risk estimates are consistent and clinically applicable for ≤5 mm UIAs selected for management without repair.

**Systematic Review Registration:**

https://www.crd.york.ac.uk/prospero/, identifier: CRD42019121522.

## Introduction

Incidental small unruptured intracranial aneurysms (ISUIA) are being increasingly discovered with greater utilization of neuroimaging. This creates significant management dilemma for both the physicians and patients, since future aneurysmal rupture causing subarachnoid hemorrhage (SAH) is associated with a risk of death, and in those who survive, a risk of cognitive impairment and disability (1, 2). This requires physicians to balance the risk of harm from preemptive UIA repair to prevent SAH and rupture risk associated with follow-up without repair.

Overall, small UIAs are considered to be at low risk of rupture based on large prior studies. The International Study of Unruptured Intracranial Aneurysms (ISUIA) (3) and the Unruptured Cerebral Aneurysm Study (UCAS) Japan (4) provide insight into understanding the future rupture risk. However, aneurysm repair in these studies was not performed at random, but targeted to UIAs that considered at greater risk of SAH. This treatment selection bias may contribute to underestimation of rupture risk during follow-up without aneurysm repair (5). In addition, aneurysm rupture risk scoring systems such as the Population, Hypertension, Age, Size, Earlier subarachnoid haemorrhage, and Site (PHASES) (6) that utilize this data are subjected to the limitations of the underlying studies.

In clinical practice, physicians consider aneurysm size as a key factor in predicting future UIA rupture (6) and commonly extrapolate a predicted annualized rupture rate across the remaining healthy lifetime of the patient when considering risk and benefit of UIA repair (7). This pragmatic approach is supported by a recent rigorous systematic review of UIAs, which included data from the ISUIA and the UCAS (8). However, mean follow-up in the included studies was <5 years and, therefore, utilization of this methodology extrapolates future rupture risk beyond the duration of observed data. In addition, extrapolation of an annualized rupture rate assumes a constant rate of rupture across the lifetime time horizon. This is also unlikely to be accurate due to potential aneurysm growth or morphological changes over the lifespan of the patient.

Therefore, we aimed to use meta-analytic methods to synthesize the proportion of rupture of ≤10 mm UIAs when selected by physicians for management without aneurysm repair. We aimed to stratify results by size threshold and also aimed to determine the level of precision in the risk estimate and to explore heterogeneity using meta-regression.

## Methods

### Protocol and Registration

This study protocol was prospectively registered with the PROSPERO (CRD42019121522). Search strategy, study selection, data extraction, risk of bias assessment, and data analysis were performed in accordance with the Preferred Reporting Items for Systematic Reviews and Meta-Analyses (PRISMA) guidelines.

### Search Strategy, Data Sources, and Data Management

The prepiloted search strategy was designed with an experienced medical librarian with an aim of high sensitivity (Supplementary Material). We searched the Ovid MEDLINE, EMBASE, Web of Science Core Collection, and the Cochrane Central Register of Controlled Trials from inception to August 2020. Reference lists of previous systematic reviews and included studies were reviewed, but gray literature sources were not included. Covidence systematic review software (Veritas Health Innovation, Melbourne, Australia) was utilized during eligibility assessment. Microsoft Excel and Endnote were utilized for data collection, data checking, and reference management.

### Study Eligibility

Studies that included participants with ≤10 mm UIAs in both the anterior and posterior circulation that did not have neurosurgical or endovascular repair and underwent follow-up were evaluated for inclusion. Randomized and non-randomized clinical trials, prospective and retrospective cohort studies, and case series with >10 patients published in English were evaluated. We included studies that reported aneurysm rupture as an outcome. When aneurysm rupture was reported as a clinical outcome in a mixed cohort of unruptured intradural and cavernous internal carotid aneurysms, we only included data if > 85% of the cohort harbored intradural aneurysms. For studies that included data published more than once, the study report with the most informative dataset was included. Two investigators independently evaluated studies for eligibility against the pre-specified inclusion criteria. Initially, titles and abstracts were screened. Full-text reports were obtained for all the potentially relevant reports and when there was ambiguity on whether or not the articles met inclusion criteria, disagreements were resolved by consensus.

### Data Abstraction

Two investigators performed independent data extraction using a pre-piloted standardized electronic datasheet that included: study author, publication year, study start and end years, country of source population, study design, number of patients, mean or median age, number of aneurysms, number of patients with multiple aneurysms, number of patients with prior SAH, mean or median follow-up, and number of aneurysms that ruptured during follow-up. When aneurysm data were stratified by size, data were extracted under the following size thresholds commonly reported in the literature: ≤10, ≤7, ≤5, and ≤3 mm. Aneurysm shape and anatomical location data were also extracted where available. Irregular aneurysms were defined as UIAs that were reported as irregular, multilobular, or with a daughter sac or aneurysm bleb. For anatomical location analysis, the total number of anterior and posterior circulation UIAs of ≤10 mm was extracted. If specific anatomical location was reported, the total number of anterior communicating artery and anterior cerebral artery aneurysms for UIAs of ≤10 mm was extracted. Discrepancies were resolved by consensus. To improve accuracy of analyses for size, we included details from additional publications of the same study cohort and emailed the corresponding authors of original publications. A separate investigator independently checked the data for consistency and completeness following double data extraction and receipt of unpublished data. An experienced investigator performed all the data analyses, which were checked independently by a separate experienced investigator.

### Risk of Bias Assessment

To assess the quality of the included non-randomized studies, the pre-piloted modified Newcastle–Ottawa Scale (NOS) (9) was used (Supplementary Material). Two investigators independently rated included studies on selection, comparability, and outcome and recorded information on each study to justify the judgment made. Any disagreements were resolved by consensus. The NOS ratings were categorized as good quality, fair quality, and poor quality based on the Agency for Healthcare Research and Quality (AHRQ) standards (10).

### Outcome

The pre-specified outcome was rupture of the index UIA included at study entry. The proportion of UIAs that ruptured was extracted from each study. Aneurysmal SAH from *de novo*, dissecting, or fusiform aneurysms was excluded. A *post-hoc* decision was made to perform a per aneurysm analysis, since one aneurysm in one patient ruptures at one time, while a patient may harbor multiple aneurysms. Aneurysm multiplicity was taken into account by extracting the total number of aneurysms for UIAs of ≤10 mm. If this was not reported, the total number of aneurysms in the ≤10 mm cohort was derived by applying the proportion of multiple aneurysms in the total observation cohort to the number of patients. If this was not reported, then patients with multiple aneurysms were assumed to harbor two UIAs. For aneurysm shape analysis, if the total number of irregular aneurysms for UIAs of ≤10 mm was not reported, the total number of aneurysms in the ≤10 mm cohort was derived by applying the same proportion of irregular aneurysms in the total observation cohort. For anatomical location analysis, if anatomical location data were not reported for UIAs of ≤10 mm, the number of aneurysms in the ≤10 mm cohort was derived by applying the same proportion of the anatomical location information in the total observation cohort. Median follow-up values were used as best estimates when mean values were missing (11). If mean follow-up was not specifically reported for the ≤10 mm cohort, the mean follow-up of the total observation cohort for all the aneurysm sizes was utilized.

### Addressing Heterogeneity

The *I*^2^ statistic was used to identify heterogeneity, which was categorized as low (<25%), moderate (25–75%), or significant (>75%) (12). Heterogeneity was explored using outlier and influence analyses. If a reasonable rationale due to clinical and/or methodological diversity was evident, the outlier study was excluded from data synthesis to reduce bias in the pooled proportion estimate and additional sensitivity analyses were performed with and without outlier studies (13).

### Meta-Analytic and Meta-Regression Methods

Random-effects data synthesis was carried out using a random intercept logistic regression model for meta-analysis of proportions. The Wilson procedure was utilized for the 95% CIs. Random-effects meta-regression was used to explore categorical or continuous covariates for residual between-study heterogeneity. Additional information is given in the Supplementary Materials. Prespecified covariates included aneurysm size and exposure to prior SAH. Additional covariates included aneurysm shape and anatomical location. Residual heterogeneity was identified using the *I*^2^ statistic. *p*-values were two-sided with values < 0.05 that are considered as statistically significant. Statistical analyses and graphical output were performed in R (version 3.6.3) using the following packages: dmetar (14), meta (15), and metafor (16).

### Sensitivity Analysis and Small-Study Effects

Prespecified sensitivity analysis was performed using size thresholds of ≤7, ≤5, and ≤3 mm. Additional *post-hoc* sensitivity analyses were performed including outlier studies, for high-quality studies, and using the leave-one-out method to confirm that the pooled rupture proportion was not driven by a single study. Reporting bias and small-study effects were examined by visual assessment of a funnel plot, calculation of Egger's regression intercept, and examination of the random-effects and fixed-effects estimates (17).

## Results

**Figure 1:**
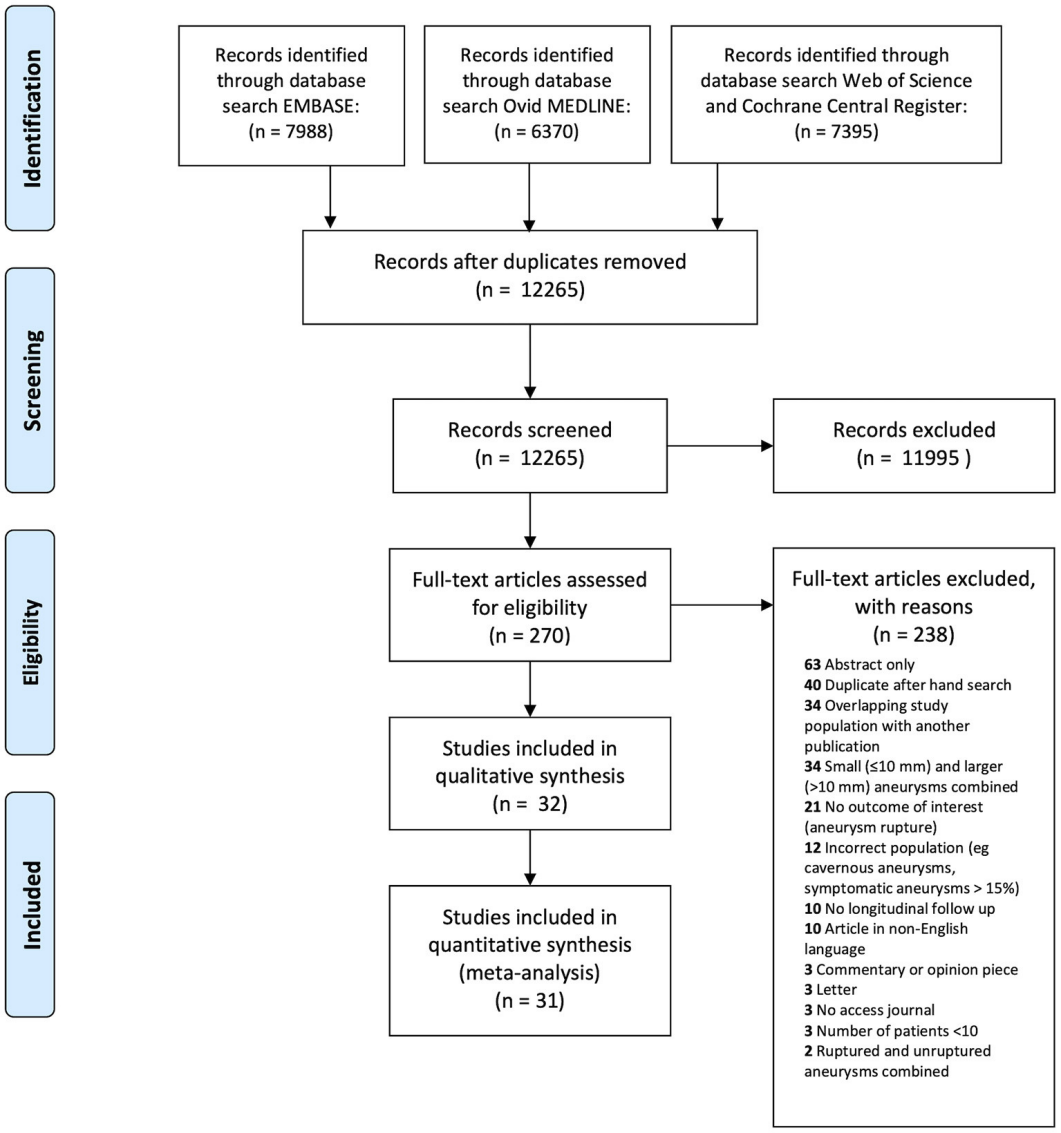
The Preferred Reporting Items for Systematic Reviews and Meta-Analyses (PRISMA) flow diagram detailing systematic search.

We identified 21,753 citations through database searches. After removing duplicates, we screened 12,265 citations for eligibility (Figure 1). After full-text review of 270 studies judged to be potentially eligible, we included 32 studies. Five study authors contributed additional unpublished data for sensitivity analyses (18–22).

### Study Characteristics

Baseline study characteristics are given in Table 1. In the 32 studies, source populations were Japan (7 studies) (23–29) United States (7 studies) (20, 22, 30–34), international (5 studies) (3, 35–38), Korea (4 studies) (39–42), Australia (2 studies) (43, 44), and one each from China (45), Netherlands (46), United Kingdom (47), Switzerland (18), Germany (48), Poland (21), and Finland (19). Majority (23 studies) were retrospective and of the prospective studies, two of the three largest studies were from Japan (26, 27). There were no randomized controlled trials. Participants were included in studies from 1956 (19) to 2019 (44). Study publication occurred between 1995 and 2020, with majority (21 studies) published in the last decade.

**Table 1:** Baseline study characteristics.

**Study**	**Study-year start**	**Study-year end**	**Source population**	**Study type**	**Mean age**	**Median age**	**No. of total patients in follow-up**	**No. of total aneurysms in follow up**	**No. of total patients with prior SAH**	**No. of total patients with multiple aneurysms**	**Mean follow-up (Years)**	**Median follow-up (Years)**	**No. of patients with ≤10 mm aneurysms**	**No. of ≤10 mm aneurysms**	**No. of patients with rupture**	**No. of patients with prior SAH and rupture**	**NOS** **quality score**
Juvela et al. (19)	1956	1978	Finland	Prospective	41.8	NA	142	181	131	33	NA	21.0	135	NA	31	30	9
Wiebers-et al. (38)	1970	1991	International	Retrospective	52.7	NA	1,449	1,937	722	364	8.3	NA	1,065	NA	18	17	8
Wiebers-et al. (3)	1991	1998	International	Prospective	55.2	NA	1,692	2,686	615	679	4.1	NA	1,049	NA	15	8	8
Sonobe et al. (28)	2000	2004	Japan	Prospective	61.9	62.0	374	448	36	124	3.4	NA	374	448	7	1	8
Molenberg et al. (46)	1998	2017	Netherlands	Retrospective	NA	56.0	206	267	82	113	NA	1.0	NA	256	0	0	8
Morita et al. (26)	2001	2004	Japan	Prospective	65.0	NA	2,998	3,647	99	824	1.7	NA	NA	3,323	62	NA	8
Murayama et al. (27)	2003	2012	Japan	Prospective	66.0	NA	1,556	1,960	44	707	3.8	NA	NA	1,921	43	NA	8
Serrone et al. (34)	2001	2012	United States	Retrospective	61.1	NA	192	234	2	73	3.2	NA	NA	194	1	0	8
So et al. (43)	1997	2006	Australia	Retrospective	51.1	NA	208	285	63	58	1.8	NA	NA	262	3	NA	8
Guresir et al. (48)	1999	2012	Germany	Retrospective	55.0	NA	263	384	0	136	4.0	NA	263	384	3	0	7
Zylkowski et al. (21)	2006	2013	Poland	Retrospective	57.8	NA	70	110	22	NA	3.0	NA	70	110	3	1	7
Irazabal et al. (32)	1989	2009	United States	Retrospective	NA	49.0	38	45	0	6	7.9	NA	38	45	0	0	7
Mizoi et al. (25)	1984	1993	Japan	Retrospective	NA	60.0	49	67	5	NA	4.3	NA	15	21	0	0	7
Burns et al. (30)	1987	2006	United States	Retrospective	64.0	NA	165	191	12	46	NA	3.9	NA	173	1	NA	7
Byoun et al. (39)	2000	2008	Korea	Retrospective	63.1	NA	NA	654	NA	NA	NA	1.6	NA	624	11	3	7
Chien et al. (22)	2005	2015	United States	Retrospective	61.8	NA	382	520	20	104	2.7	NA	NA	490	6	NA	7
Matsumoto et al. (24)	1996	2008	Japan	Retrospective	65.0	NA	111	136	8	29	NA	NA	NA	113	3	NA	7
Teo and George (47)	NA	NA	United Kingdom	Retrospective	53.0	NA	94	152	30	NA	3.4	NA	NA	126	3	0	7
Jeon et al. (41)	2001	2011	Korea	Retrospective	59.4	NA	524	568	0	NA	2.9	NA	524	568	2	0	6
Huang et al. (44)	2011	2019	Australia	Retrospective	NA	73.3	193	255	13	83	NA	4.9	185	243	0	0	6
Choi et al. (40)	2007	2010	Korea	Retrospective	57.4	NA	135	173	21	NA	6.1	6.0	135	173	1	NA	6
Jiang et al. (45)	2007	2012	China	Retrospective	52.3	NA	40	50	0	NA	3.0	NA	40	50	0	0	6
Gondar et al. (18)	2006	2014	Switzerland	Prospective	55.1	NA	292	368	17	93	3.2	NA	NA	368	3	1	6
Broderick et al. (36)	NA	NA	International	Prospective	51.4	NA	113	148	0	NA	NA	NA	108	NA	2	0	5
Tsutsumi et al. (29)	1976	1997	Japan	Retrospective	70.8	NA	62	83	0	14	4.3	NA	56	NA	3	0	5
Bor et al. (35)	NA	NA	International	Prospective	55.0	NA	363	468	98	163	2.8	2.1	NA	403	3	NA	5
Tsukahara et al. [37	1999	2001	International	Retrospective	NA	NA	181	209	38	NA	NA	NA	NA	144	5	0	5
Loumiotis et al. (33)	2008	2011	United States	Prospective	64.4	NA	125	160	0	22	1.4	1.3	125	160	0	0	4
Gibbs et al. (31)	1989	2002	United States	Retrospective	47.9	NA	21	22	0	1	7.7	NA	21	22	0	0	4
Matsubara et al. (23)	NA	NA	Japan	Retrospective	62.8	NA	140	166	5	51	1.5	NA	NA	158	0	0	4
Oh et al. (42)	2007	2008	Korea	Retrospective	72.2	NA	19	19	0	0	2.0	NA	19	19	0	0	3
Wilkinson et al. (20)	2000	2016	United States	Retrospective	52.0	NA	17	22	2	NA	NA	5.3	17	22	0	0	3

*SAH, subarachnoid hemorrhage*.

Across the 32 studies, a total of 12,214 participants with 16,615 UIAs were managed without repair across all the size strata. Of these, 13,966 were ≤10 mm UIAs with mean follow-up of 4.3 years (range 1.4–21 years). Overall, 70.8% of participants were female; mean age was 58.3 ± 7.1 years. The mean proportion of included patients included with prior SAH was 14.5% (range 0–92.3%).

Twelve studies were judged of the good AHRQ standard with lower risk of bias (Table 2). Only one study scored three stars in the outcome domains having complete follow-up (19).

**Table 2:** Risk of bias assessment.

	**Selection**				**Comparability**	**Outcome**				
**Study**	**Representativeness of the exposed cohort**	**Selection of the non-exposed cohort**	**Ascertainment of prior subarachnoid hemorrhage**	**Demonstration that outcome of interest was not present at start of study**	**Comparability of cohorts on the basis of the design or analysis**	**Assessment of outcome**	**Was follow-up long enough for outcomes to occur?**	**Adequacy of follow-up of cohorts**	**NOS quality score**	**AHRQ standards**
**AHRQ good standard**								
Juvela et al. (19)	*	*	*	*	**	*	*	*	9	Good
Molenberg et al. (46)	*	*	*	*	**	*		*	8	Good
Morita et al. (26)	*	*	*	*	**	*		*	8	Good
Murayama et al. (27)	*	*	*	*	**	*		*	8	Good
Serrone et al. (34)	*	*	*	*	**	*		*	8	Good
So et al. (43)	*	*	*	*	**	*		*	8	Good
Sonobe et al. (28)	*	*	*	*	**	*		*	8	Good
Wiebers- et al. (38)	*	*	*	*	**	*		*	8	Good
Wiebers- et al. (3)	*	*	*	*	**	*		*	8	Good
Irazabal et al. (32)		*	*	*	**	*		*	7	Good
Matsumoto et al. (24)	*	*		*	**	*		*	7	Good
Mizoi et al. (25)	*	*		*	**	*		*	7	Good
**AHRQ fair standard**								
Huang et al. (44)		*		*	**	*		*	6	Fair
Jeon et al. (41)			*	*	**	*		*	6	Fair
Jiang et al. (45)		*		*	**	*		*	6	Fair
Tsutsumi et al. (29)			*	*	*	*		*	5	Fair
**AHRQ poor standard**								
Burns et al. (30)	*	*	*	*	**	*			7	Poor
Byoun et al. (39)	*	*	*	*	**	*			7	Poor
Chien et al. (22)	*	*	*	*	**	*			7	Poor
Guresir et al. (48)	*	*	*	*	**			*	7	Poor
Teo and George (47)	*	*	*	*	**	*			7	Poor
Zylkowski et al. (21)	*	*	*	*	**	*			7	Poor
Choi et al. (40)	*	*	*	*	*			*	6	Poor
Gondar et al. (18)	*	*		*	**			*	6	Poor
Bor et al. (35)	*	*	*	*	*				5	Poor
Broderick et al. (36)			*	*	**	*			5	Poor
Tsukahara et al. (37)	*	*		*	**				5	Poor
Gibbs et al. (31)				*	**			*	4	Poor
Loumiotis et al. (33)				*	**			*	4	Poor
Matsubara et al. (23)		*		*	**				4	Poor
Oh et al. (42)				*		*		*	3	Poor
Wilkinson et al. (20)					**	*			3	Poor

*Thresholds for converting the Newcastle–Ottawa scales to the Agency for Healthcare Research and Quality (AHRQ) standards (good, fair, and poor):(10) Good quality: 3 or 4 stars in selection domain and 1 or 2 stars in comparability domain and 2 or 3 stars in outcome/exposure domain*.

*Fair quality: 2 stars in selection domain and 1 or 2 stars in comparability domain and 2 or 3 stars in outcome/exposure domain*.

*Poor quality: 0 or 1 star in selection domain or 0 star in comparability domain or 0 or 1 star in outcome/exposure domain*.

### Data Synthesis

Preliminary data synthesis included 32 studies with 229 rupture events in 13,966 ≤10 mm UIAs over a mean of 4.3 years with significant heterogeneity (*I*^2^ = 85%) (Supplementary Figure 1). Outlier analysis identified an outlier (19), with a study rupture proportion of 18.7% (95% CI 13.5–25.3) compared to the pooled proportion of 1.1% (95% CI 0.7–1.7). With leave-one-out analysis, this was the only study that contributed greatly to heterogeneity, with *I*^2^ reduction from 85 to 53% (Supplementary Figure 2). Further outlier and influence diagnostics also confirmed these findings (Supplementary Figures 3, 4).

Examination of the outlier study (19) revealed underlying clinical and methodological diversity responsible for between-study heterogeneity. All the patients in this study were managed without aneurysm repair due to lack of treatment availability. In the remaining 31 studies, treatment was considered and a clinical decision made to manage the patient without aneurysm repair. In addition, the outlier study had the longest follow-up (21 vs. mean 3.7 years), the highest proportion of exposure to prior SAH (92.3 vs. mean 11.9%) and an earlier recruitment period (1956–1978 vs. median midpoint 2006). A *post-hoc* decision was made to exclude this outlier study (19) from the main analysis because of lack of availability of aneurysm repair at the time of the study and to appropriately address heterogeneity (13).

### Meta-Analysis and Meta-Regression

**Figure 2:**
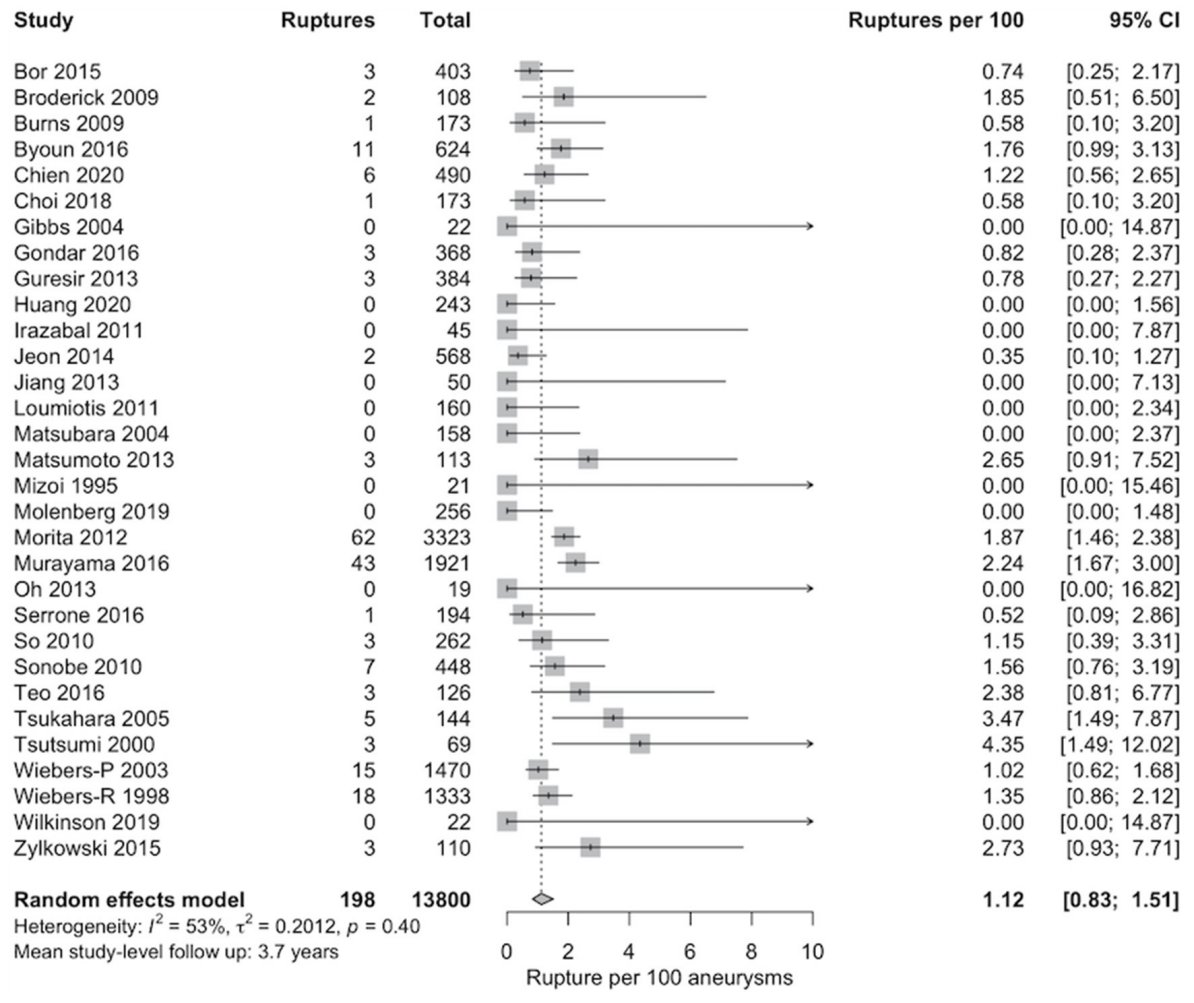
Pooled proportion of rupture per 100 unruptured intracranial aneurysms (UIAs) ≤10 mm managed without aneurysm repair. Random-effects data synthesis was carried out using a random intercept logistic regression model. The Wilson procedure was utilized for calculation of the 95% CIs.

The meta-analytic proportion of ruptures without aneurysm repair was synthesized across the remaining 31 studies and included 198 rupture events in 13,800 ≤10 mm UIAs (Figure 2). Aneurysm rupture occurred in 1.1% of ≤10 mm UIAs (95% CI 0.8–1.5; *I*^2^ = 53%) over mean 3.7 years of follow-up.

Meta-regression included the following study-level covariates: proportion of patients with exposure to prior SAH, proportion with aneurysm size ≤5 mm, proportion with multiple aneurysms, proportion with irregular aneurysms, proportion with posterior circulation aneurysms, proportion with anterior circulation aneurysms, proportion with anterior communicating artery or anterior cerebral aneurysms, mean age, source population categorized as Japanese or non-Japanese, study type categorized as prospective or retrospective, and length of follow-up.

In subgroup analysis according to whether or not the source population was Japanese, a larger proportion of ruptures was identified in Japanese compared to non-Japanese populations during follow-up [test for subgroup differences, *p* < 0.001, residual *I*^2^ = 0% (95% CI 0.0–19.4%)] (Supplementary Figure 5). However, the mean age in Japanese samples (64.5 years) was greater than in non-Japanese samples (57.4 years, *p* = 0.001).

Associations between other covariates in the remaining meta-regression analyses including proportion with prior SAH, proportion with ≤5 mm aneurysms, proportion with multiple aneurysms, proportion with irregular aneurysms, proportion with posterior circulation aneurysms, proportion with anterior circulation aneurysms, proportion with anterior communicating artery or anterior cerebral aneurysms, study type, study-level age as a continuous variable, and follow-up time as a continuous variable were not detected (Supplementary Figures 6–15).

### Sensitivity Analysis and Small-Study Effects

In leave-one-out sensitivity analysis, the meta-analytic proportion of ruptures remained consistent with no clinically relevant effect (Supplementary Figure 16). Heterogeneity could be further reduced by exclusion of Murayama et al. (27) (residual *I*^2^ = 42%) or Jeon et al. (41) (residual *I*^2^ = 44%), but these levels of reduction in heterogeneity were considered unimportant.

Findings were consistent in prespecified sensitivity analyses at all the size thresholds (Supplementary Figures 17–19). For ≤7 mm UIAs managed without repair, data were synthesized across 28 studies, 134 rupture events, and 11,371 UIAs: rupture occurred in 0.9% UIAs (95% CI 0.7–1.3; *I*^2^ = 49.1%) over 3.6 years of follow-up. For ≤5 mm UIAs managed without repair, data synthesis occurred across 24 studies, 74 rupture events, and 7,280 UIAs: rupture occurred in 1.0% UIAs (95% CI 0.8–1.3; *I*^2^ = 0%) over 3.5 years of follow-up. For ≤3 mm UIAs managed without repair, data synthesis occurred across 18 studies, 10 rupture events, and 1,228 UIAs: rupture occurred in 0.8% UIAs (95% CI 0.4–1.5; *I*^2^ = 0%) over 3.8 years of follow-up.

There were also consistent results in *post-hoc* sensitivity analyses that included the outlier study (Supplementary Figure 20). Notably, in sensitivity analyses limited to high-quality studies, the pooled proportion of rupture was greater, 1.8% (95% CI 1.5–2.0; *I*^2^ = 0%) over 3.9 years (Supplementary Figure 21).

Funnel plot distribution was asymmetrical on visual assessment and confirmed on Egger's test of the intercept (Supplementary Figure 22). Multiple possible sources of asymmetry other than random chance were identifiable. These include non-reporting bias, selective reporting bias, small-study effects, and residual heterogeneity. The presence of small-study effects was investigated with additional *post-hoc* sensitivity analysis considering a fixed-effects model estimate with no clinically relevant effect on the meta-analytic proportion of ruptures (Supplementary Figure 23).

## Discussion

In this meta-analysis of 31 studies with 13,800 UIAs 10 mm and less in size managed conservatively without repair, 0.8 to 1.5% UIAs ruptured over 3.7 years. This is an evidence-based clinically relevant pooled risk estimate with consideration of the level of precision over the observed follow-up time. Importantly, the risk estimates were consistent across studies when stratified by size for 2–5 mm UIAs. In higher quality studies, a greater proportion (1.5 to 2.0%) of ruptures were identified over a similar time period, indicating that inclusion of studies with higher risk of bias leads to underestimation of the proportion of rupture.

In addition, in our analysis of over 1,200 ≤3 mm UIAs managed conservatively without repair, we have found a rupture risk of between 0.4 and 1.5% over 3.8 years. This is similar to 0.8 to 1.3% rupture risk over 3.5 years for over 7,000 ≤5 mm UIAs. These findings are in contrast to the conclusions of ISUIA that the 5-year cumulative rupture rate of anterior circulation aneurysms <7 mm was 0% (3) and the prior systematic review and narrative synthesis (8), which analyzed 7 studies and concluded that the estimated annualized rupture rate was 0% for ≤3 mm UIAs. Subgroup analysis in this ≤3 mm UIA cohort is limited due to lack of reporting of aneurysm-related characteristics such as morphology in all the included studies. However, anatomical location for ≤3 mm ruptured aneurysm was reported for 5 of ten patients. Four ≤3 mm UIAs that ruptured were in the anterior circulation, with 3 at the anterior communicating artery or distal anterior cerebral artery. These findings are concordant with expert physician experience and recent literature that <5 mm UIAs represent a large proportion of all the ruptured aneurysms (49).

For ≤10 mm UIAs managed conservatively without repair, we did not identify anatomical location in the anterior or posterior circulation or involving the anterior communicating artery or anterior cerebral artery as sources of heterogeneity modifying the rupture risk. Majority of UIAs included were 2–5 mm and these findings are similar to the prior prospective Small Unruptured Intracranial Aneurysm Verification Study that was limited to <5 mm UIAs (28). A minority of studies reported data on aneurysm shape and our aggregate data meta-analysis did not identify irregular aneurysm shape as a source of heterogeneity. Individual participant data meta-analysis would reduce heterogeneity and increase the statistical power to better explore these associations of rupture risk with aneurysm shape and anatomical location. This is particularly important for expert physicians to consider, as irregular UIAs and anterior communicating artery and posterior communicating artery location SIUAs were associated with a higher risk of rupture compared to regular UIA shape and alternate anatomical locations in a large prospective cohort (4).

Compared to the prior systematic review, we have synthesized a meta-analytic rupture risk estimate and explored heterogeneity. To improve clinical applicability compared to the prior systematic review, studies with a high proportion of cavernous UIAs (50, 51) were excluded from this study, since they are known to have negligible risk of SAH. In addition, our broader search strategy design yielded 15 additional studies (20, 22, 30–34, 37, 38, 40, 42–46), a third of which have published (20, 22, 40, 44, 46) since the prior systematic review.

This primary study outcome of a cumulative incidence of UIA rupture over the included follow-up time period is a pragmatic method to communicate rupture risk for both the physicians and patients. This is an alternate approach to extrapolating an annualized rupture rate (8, 26) over the remaining lifetime when considering risk and benefit of UIA repair (5). Extrapolation of an annualized rupture rate is unlikely to be accurate due to multiple assumptions. First, extrapolation assumes a constant rupture rate of UIAs, which is unlikely to be plausible, since UIA growth or morphological change over time is associated with rupture (52, 53) and the risk of rupture may decrease after a certain follow-up time within the lifetime (19). Moreover, additional competing risks such as death from causes other than SAH need to be considered (5) and appropriate external data sources to assess validity of such extrapolation are lacking (54).

Overall, our results are considered applicable to pooled estimates of rupture risk for 2–5 mm UIAs managed conservatively without repair over mean of 3.7 years. This is based on the characteristics of the included cohorts: UIAs included were mostly ≤5 mm, five largest studies did not include UIAs <2 mm, and majority of studies had <5 years of follow-up. This is a clinically relevant population, since many patients with 2–5 mm UIAs are usually considered for follow-up without repair (5).

To better understand rupture risk beyond the mean of 3.7 years, additional long-term data are required. There remains only one almost lifelong prospective follow-up study of rupture risk not subjected to treatment selection bias (19). However, outcomes from this long-term follow-up cohort are not generalizable to patients with incidental UIAs identified by neuroimaging today. Only 5 of 142 patients included in this long-term follow-up cohort harbored an incidental UIA and the comorbidity profile (70% smokers, 36% hypertensive, and 21% alcohol abuse) reflects the recruitment period (1956 to 1978) in Finland at that time (55).

The main limitation of our systematic review and meta-analysis is the utilization of aggregate data and, thus, adjustment could not be made for individual patient-level factors and aneurysm-level factors, which would only be possible in an individual patient-level meta-analysis.

In addition to UIA anatomical location and shape, there are additional patient-level variables and aneurysm-level variables that would be useful to explore in future studies including smoking status, hypertension, and systolic blood pressure or aneurysm morphological factors such as aspect ratio. Regardless, this study results remain valid, since aneurysm size is a key factor consistently associated with rupture risk (6) and addition of patient-level variables and aneurysm-level variables would help to improve the precision of risk prediction within the upper and lower limits of the 95% CIs that we have already identified.

In addition, functional outcome and mortality after rupture could not be determined due to inconsistent and non-reporting of clinical outcomes. The pooled risk estimate over time was limited by case follow-up in the individual study cohorts. A third of included studies introduced bias due to inadequate follow-up identified during quality assessment.

These limitations were reduced by inclusion of a large number of studies, large number of rupture events, and careful categorization of UIAs.

## Conclusion

Our meta-analysis demonstrates that for every 1,000 UIAs that are ≤10 mm in size and selected for conservative management without repair, between 8 and 15 UIAs are estimated to rupture over 3.7 years. Pooled rupture risk estimates stratified by UIA size are consistent and clinically applicable for 2–5 mm UIAs. This is an evidence-based pragmatic method to communicate rupture risk for both the physicians and patients. To better understand and individualize long-term UIA rupture risk with greater precision, additional UIA follow-up data are required.

## Data Availability Statement

The original contributions presented in the study are included in the article/Supplementary Material, further inquiries can be directed to the corresponding author/s.

## Author Contributions

RC and TP contributed to the conception and design of the study. RC carried out the systematic search. Data abstraction was performed independently by RC and JM. NC independently checked the data abstraction. NC and L-AS independently carried out the quality and bias assessments. Statistical analysis was performed by RC and TP. RC completed the first draft of the manuscript with further additions by LL and TP. All authors contributed to data interpretation and subsequent revisions and approved the final version of the manuscript.

## Conflict of Interest

The authors declare that the research was conducted in the absence of any commercial or financial relationships that could be construed as a potential conflict of interest.

## Publisher's Note

All claims expressed in this article are solely those of the authors and do not necessarily represent those of their affiliated organizations, or those of the publisher, the editors and the reviewers. Any product that may be evaluated in this article, or claim that may be made by its manufacturer, is not guaranteed or endorsed by the publisher.
