# Supplementary material for: A Meta-Analysis of Rupture Risk for Intracranial Aneurysms 10 mm or Less in Size Selected for Conservative Management Without Repair
Source: Front Neurol. 2022 Feb 17;12:743023. doi: 10.3389/fneur.2021.743023 (PMC8893017; doi:10.3389/fneur.2021.743023)
Supplement: Supplementary file 1 [file Data_Sheet_1.docx]

SUPPLEMENTARY MATERIAL

A Meta-analysis of Rupture Risk for intracranial aneurysms 10 mm or less in size selected for conservative management without repair

Ronil V. Chandra^1,2*^, Julian Maingard^1^, Lee-Anne Slater^1,2^, Nicholas K. Cheung^1^, Leon T. Lai^2,3^, Seana L. Gall^4,5^, Amanda G. Thrift^5^, Thanh G. Phan^5,6^

^1^NeuroInterventional Radiology, Monash Medical Centre, Monash Health, Melbourne, Victoria, Australia^2^, School of Clinical Sciences at Monash Health, Monash University, Melbourne, Victoria, Australia^3^, Department of Neurosurgery, Monash Medical Centre, Monash Health, Melbourne, Victoria, Australia^4^, Menzies Institute for Medical Research, University of Tasmania, Tasmania, Australia^5^, Stroke and Ageing Research, Department of Medicine, School of Clinical Sciences at Monash Health, Monash University, Melbourne, Victoria, Australia^6^, Department of Neurology, Monash Medical Centre, Monash Health, Melbourne, Victoria, Australia

* Correspondence: Ronil V. Chandra ronil.chandra@monash.edu

Table of Contents

[Supplementary Methods - Search strategy 3](#_Toc89012537)

[Supplementary Methods - Risk of Bias Assessment 4](#_Toc89012538)

[Modified Newcastle-Ottawa Scale Scoring Sheet 4](#_Toc89012539)

[Supplementary Methods - Statistical methods 6](#_Toc89012540)

[Meta-analytic methods 6](#_Toc89012541)

[Confidence intervals 6](#_Toc89012542)

[Meta-regression 6](#_Toc89012543)

[Supplementary Results 7](#_Toc89012544)

[Supplementary Figure 1. Preliminary data synthesis of proportion of rupture per 100 aneurysms managed conservatively without repair across studies identified by systematic search. 7](#_Toc89012545)

[Supplementary Figure 2. Leave-one-out analysis of studies identified by systematic search ordered by impact on I^2^ on the pooled proportion of rupture. 8](#_Toc89012546)

[Supplementary Figure 3. Baujat diagnostic plot of the studies identified by systematic search. 9](#_Toc89012547)

[Supplementary Figure 4. Outlier and influence diagnostic plots of the studies identified by systematic search. 10](#_Toc89012548)

[Supplementary Figure 5. Sub-group analysis of pooled proportion of rupture per 100 unruptured intracranial aneurysms (UIAs) ≤10mm managed conservatively without repair with source population categorised as Japanese or non-Japanese. 11](#_Toc89012549)

[Supplementary Figure 6. Meta-regression of proportion of patients included with exposure to prior SAH as a continuous variable on proportion of rupture per 100 UIAs ≤10mm managed conservatively without repair. 12](#_Toc89012550)

[Supplementary Figure 7. Meta-regression of proportion of patients included with ≤5mm aneurysms as a continuous variable of proportion of rupture per 100 UIAs ≤10mm managed conservatively without repair. 13](#_Toc89012551)

[Supplementary Figure 8. Meta-regression of proportion of patients included with multiple aneurysms as a continuous variable on proportion of rupture per 100 UIAs ≤10mm managed conservatively without repair. 14](#_Toc89012552)

[Supplementary Figure 9. Meta-regression of proportion of patients included with irregular shaped aneurysms as a continuous variable on proportion of rupture per 100 UIAs ≤10mm managed conservatively without repair. 15](#_Toc89012553)

[Supplementary Figure 10. Meta-regression of proportion of patients included with posterior circulation aneurysms as a continuous variable on proportion of rupture per 100 UIAs ≤10mm managed conservatively without repair. 16](#_Toc89012554)

[Supplementary Figure 11. Meta-regression of proportion of patients included with anterior circulation aneurysms as a continuous variable on proportion of rupture per 100 UIAs ≤10mm managed conservatively without repair. 17](#_Toc89012555)

[Supplementary Figure 12. Meta-regression of proportion of patients included with anterior communicating artery or anterior cerebral aneurysms as a continuous variable on proportion of rupture per 100 UIAs ≤10mm managed conservatively without repair. 18](#_Toc89012556)

[Supplementary Figure 13. Meta-regression of age as a continuous variable on proportion of rupture per 100 UIAs ≤10mm managed conservatively without repair. 19](#_Toc89012557)

[Supplementary Figure 14. Sub-group analysis of study type per 100 UIAs ≤10mm managed conservatively without repair categorised as prospective or retrospective. 20](#_Toc89012558)

[Supplementary Figure 15. Meta-regression of follow up time as a continuous variable on proportion of rupture per 100 UIAs ≤10mm managed conservatively without repair. 21](#_Toc89012559)

[Supplementary Figure 16. Sensitivity analysis using leave-one-out method ordered by impact on the pooled proportion of rupture with residual *I^2^* indicated. 22](#_Toc89012560)

[Supplementary Figure 17. Sensitivity analysis using ≤7mm UIA size threshold. 23](#_Toc89012561)

[Supplementary Figure 18. Sensitivity analysis using ≤5mm UIA size threshold. 24](#_Toc89012562)

[Supplementary Figure 19. Sensitivity analysis using ≤3mm UIA size threshold. 25](#_Toc89012563)

[Supplementary Figure 20. Sensitivity analysis including outlier study. 26](#_Toc89012564)

[Supplementary Figure 21. Sensitivity analysis limited to good standard studies. 27](#_Toc89012565)

[Supplementary Figure 22. Funnel plot to examine small-study effects included in meta-analysis. 28](#_Toc89012566)

[Supplementary Figure 23. Sensitivity analysis to consider small-study effects. 29](#_Toc89012567)

# Supplementary Methods - Search strategy

**MEDLINE**

1. Intracranial Aneurysm/
2. ((basilar or berry or brain or cerebral or intracranial or cerebrovascular) adj5 aneurysm*).ti,ab.
3. (saccular or unruptured or incidental or asymptomatic).ti,ab.
4. 1 or 2
5. 3 and 4

**EMBASE**

1. intracranial aneurysm/ or brain artery aneurysm/ or unruptured intracranial aneurysm/
2. ((basilar or berry or brain or cerebral or intracranial or cerebrovascular) adj5 aneurysm*).ti,ab.
3. 1 OR 2
4. (saccular or unruptured or incidental or asymptomatic).ti,ab.
5. 3 AND 4

**COCHRANE CENTRAL**

1. Intracranial Aneurysm/
2. ((basilar or berry or brain or cerebral or intracranial or cerebrovascular) adj5 aneurysm*).ti,ab.
3. (saccular or unruptured or incidental or asymptomatic).ti,ab.
4. 1 or 2
5. 3 and 4

**WEB OF SCIENCE**

1. TOPIC: (((basilar or berry or brain or cerebral or intracranial or cerebrovascular) NEAR/4 aneurysm*))
2. TOPIC:((saccular or unruptured or incidental or asymptomatic))
3. 1 AND 2

# Supplementary Methods - Risk of Bias Assessment

## Modified Newcastle-Ottawa Scale Scoring Sheet

*Prior subarachnoid haemorrhage is the exposure of interest*

*Aneurysmal rupture is the outcome of interest*

**Please BOLD AND UNDERLINE YOUR ANSWER**

***Please write 1-2 sentences to explain your rationale***

**First Author name:**

**Publication year:**

**Selection**

1. *Representativeness of the exposed cohort (with prior subarachnoid haemorrhage)*

Is the included cohort

- 1. truly representative of the average population with small (≤10 mm) cerebral aneurysms in the community*
  2. somewhat representative of the average population with small (≤10 mm) cerebral aneurysms in the community*
  3. selected group of cerebral aneurysm patients
  4. no description of the derivation of the cohort

1. *Selection of the non-exposed cohort (without prior subarachnoid haemorrhage)*

Is the cohort without prior SAH drawn from the same community?

- 1. drawn from the same community as the exposed cohort*
  2. drawn from a different source
  3. no description of the derivation of the non-exposed cohort

1. *Ascertainment of prior subarachnoid haemorrhage*

How was prior subarachnoid haemorrhage determined?

- 1. secure record (e.g. medical or surgical records)*
  2. patient medical consultation or interview*
  3. written patient self-report
  4. no description

1. *Demonstration that outcome of interest (aneurysmal rupture of observed aneurysm) was not present at start of study*

Was the observed aneurysm not ruptured at study entry?

- 1. yes*
  2. no

**Comparability**

1. *Comparability of cohorts on the basis of the design or analysis*

Does the study consider known associations or predictors?

- 1. study controls and/or reports outcomes for stratified aneurysm sizes under 10 mm*
  2. study controls and/or reports outcomes for any additional factor (e.g. hypertension or smoking status or aneurysm morphology or aneurysm location)*

**Outcome**

1. *Assessment of outcome*

How was the rupture of the observed aneurysm verified?

- 1. Medical imaging*
  2. Medical record (including surgical or death records) or record data linkage*
  3. patient self-report
  4. no description

1. *Was follow-up long enough for outcomes to occur*, i.e. mean follow up is >12 YEARS?
   1. Yes*
   2. No
2. *Adequacy of follow up of cohorts*

How complete was the follow up?

- 1. Complete follow up - all subjects accounted for*
  2. Small number lost to follow up unlikely to introduce bias— with >90% follow up or description provided for all those lost to follow up*
  3. Follow up rate < 90% and no description of those lost
  4. No statement regarding completeness of follow up

# Supplementary Methods - Statistical methods

## Meta-analytic methods

We used a random intercept logistic regression model for statistical procedures and data synthesis, a type of generalised linear mixed model.(1, 2) This model is an exact likelihood model based on the binomial distribution; has greater accuracy of the logit proportion and coverage percentages of the corresponding confidence intervals compared to standard normal approximation logit transformation, especially when there are small sample sizes and large between-study heterogeneity.(3) The risk of sparse data bias is minimised since no continuity correction is required. The maximum-likelihood method is utilised to estimate between-study variance τ^2^. The limitation of this statistical approach is that individual study weights utilised to pool the individual studies to create the pooled rupture risk estimate will not be available. We have utilised the Knapp–Hartung adjustment as recommended by the Cochrane collaboration.(4, 5)

## Confidence intervals

The Wilson procedure was used for calculation of the confidence intervals (CI). The derived CIs more accurately reflect the true CI with less variability compared to the Clopper-Pearson method, particularly when samples are small. The Wilson procedure is suggested for extreme proportions and can be utilised for all sample sizes.(6, 7)

## Meta-regression

Meta-regression using a random intercept logistic regression model was utilised to explore single categorical or continuous covariates for between-study heterogeneity. Risks of false positive findings have been minimised by using random-effects meta-regression, use of a single covariate with >20 studies in each model, avoiding multivariable meta-regression, and using the Knapp-Hartung adjustment.(8-10) In addition, all meta-regression analyses were considered exploratory investigations of observational associations across studies, and hypothesis generating to guide further research effort.(11)

# Supplementary Results

## Supplementary Figure 1. Preliminary data synthesis of proportion of rupture per 100 aneurysms managed conservatively without repair across studies identified by systematic search.


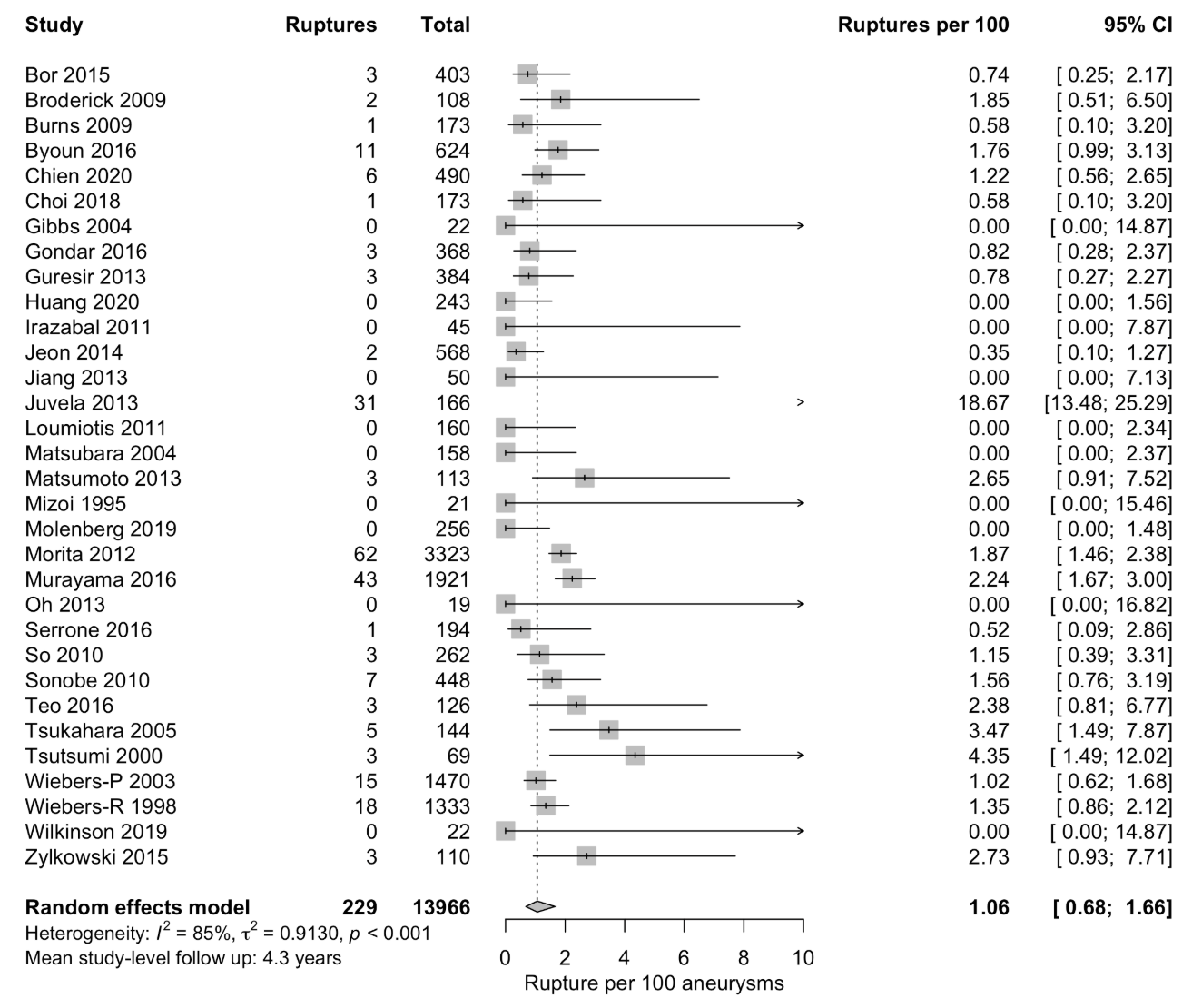


## Supplementary Figure 2. Leave-one-out analysis of studies identified by systematic search ordered by impact on I^2^ on the pooled proportion of rupture.


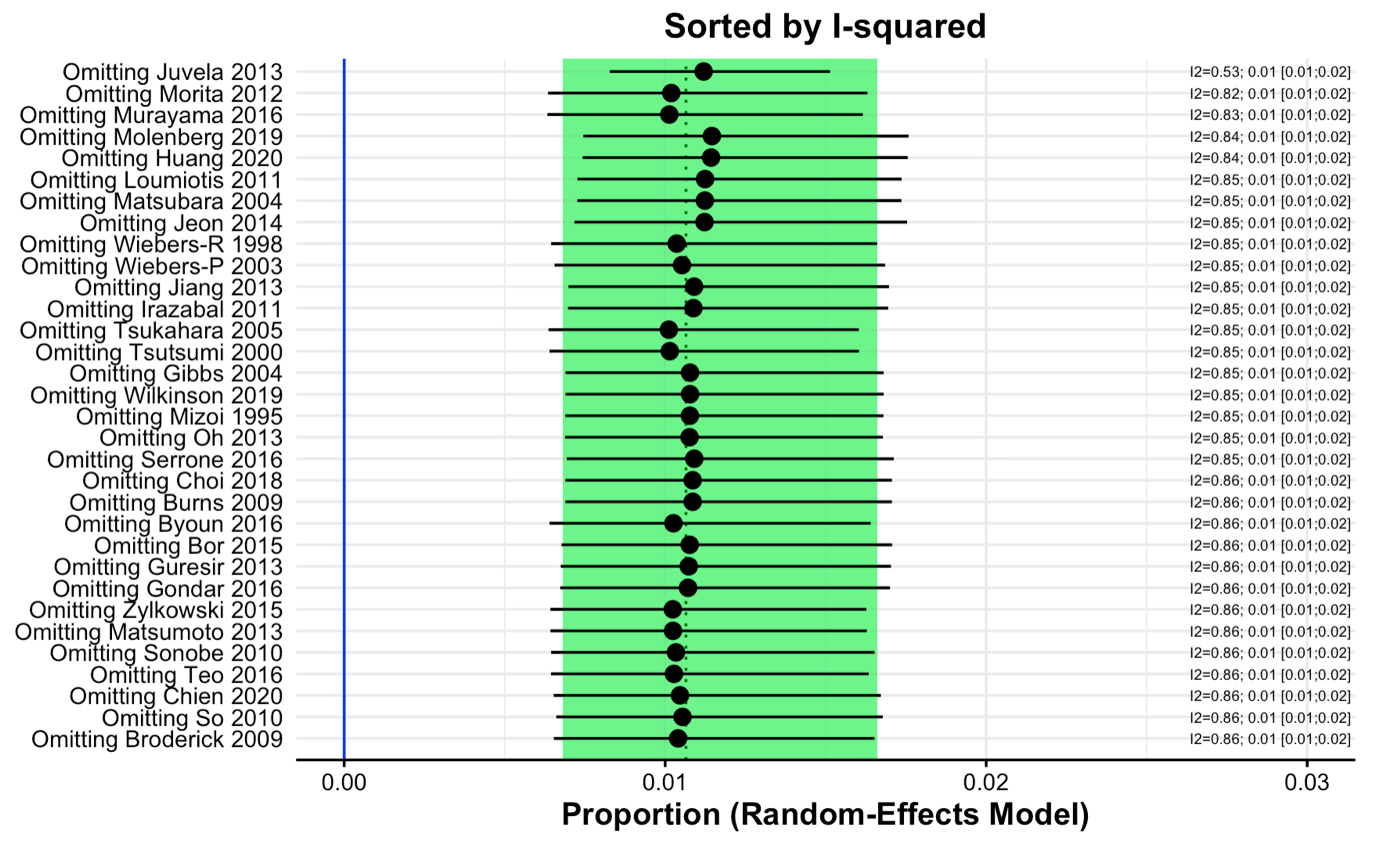


A single study(12) contributes disproportionately to heterogeneity and reduces the precision of the pooled proportion of rupture.

## Supplementary Figure 3. Baujat diagnostic plot of the studies identified by systematic search.


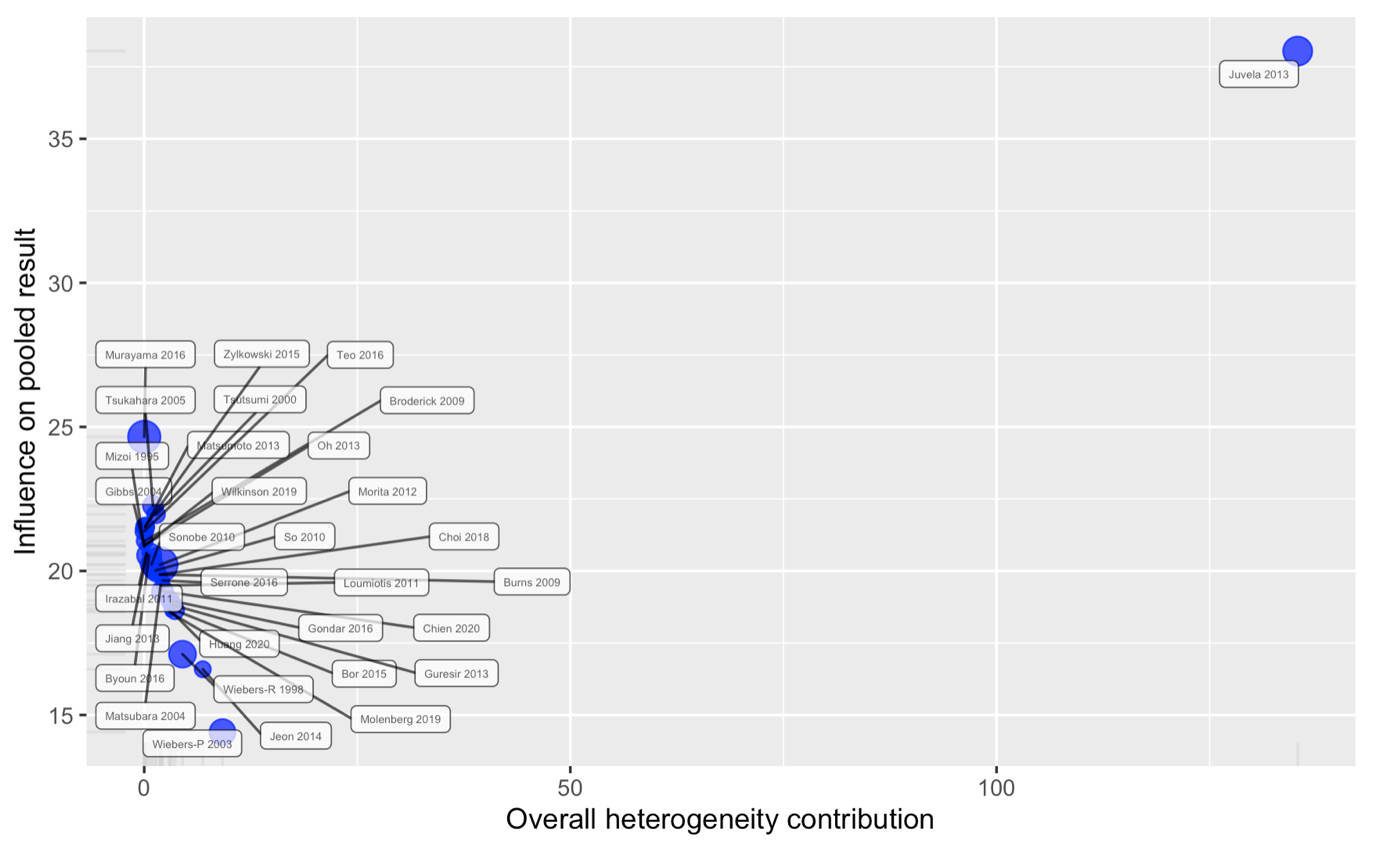


A single study(12) contributes disproportionately to heterogeneity and influences the pooled proportion of rupture.

## Supplementary Figure 4. Outlier and influence diagnostic plots of the studies identified by systematic search.


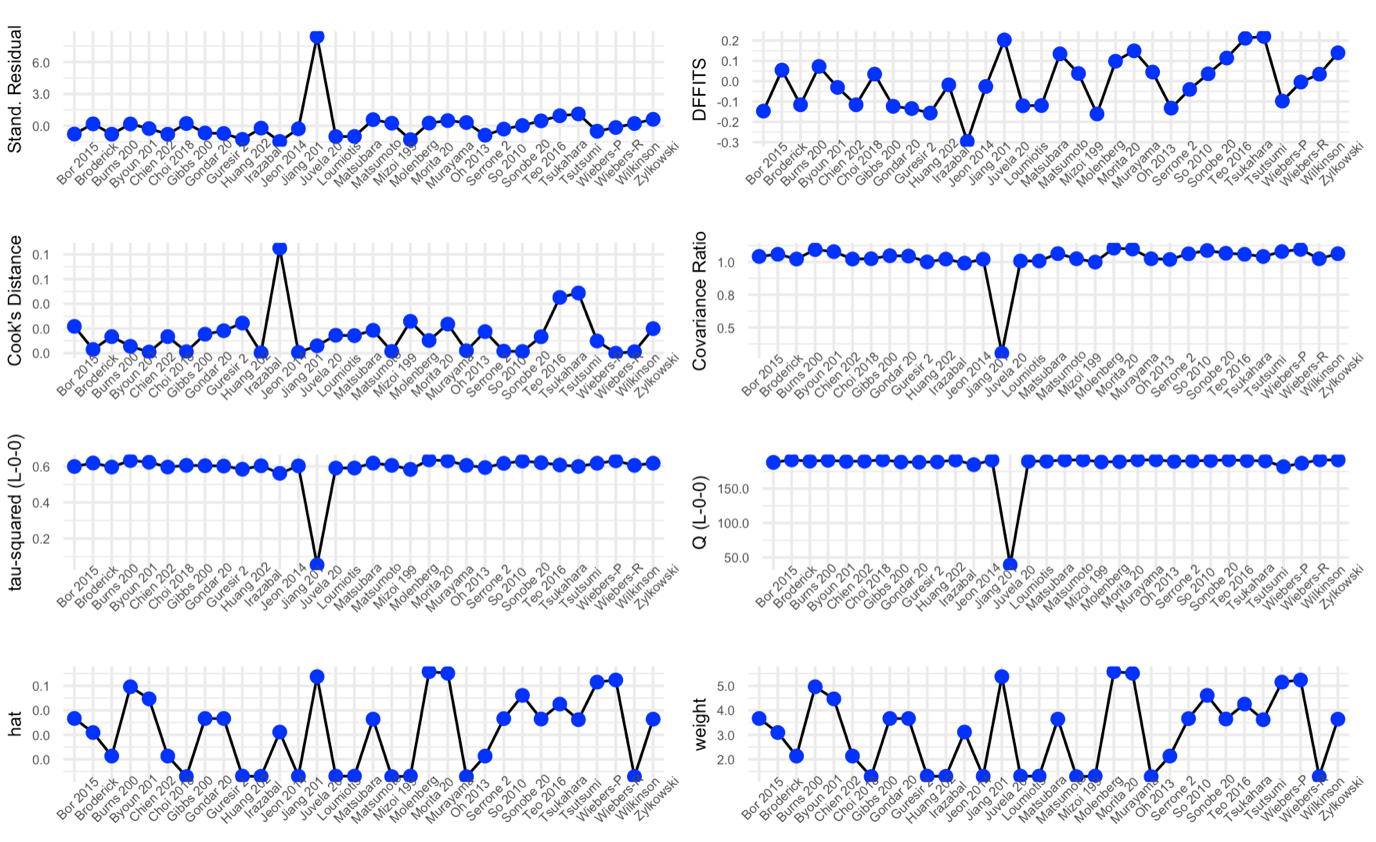


Multiple plots confirm that a single study(12) is an outlier, influences the pooled result, and reduces the precision of the estimate of the pooled proportion of rupture.

## Supplementary Figure 5. Sub-group analysis of pooled proportion of rupture per 100 unruptured intracranial aneurysms (UIAs) ≤10mm managed conservatively without repair with source population categorised as Japanese or non-Japanese.


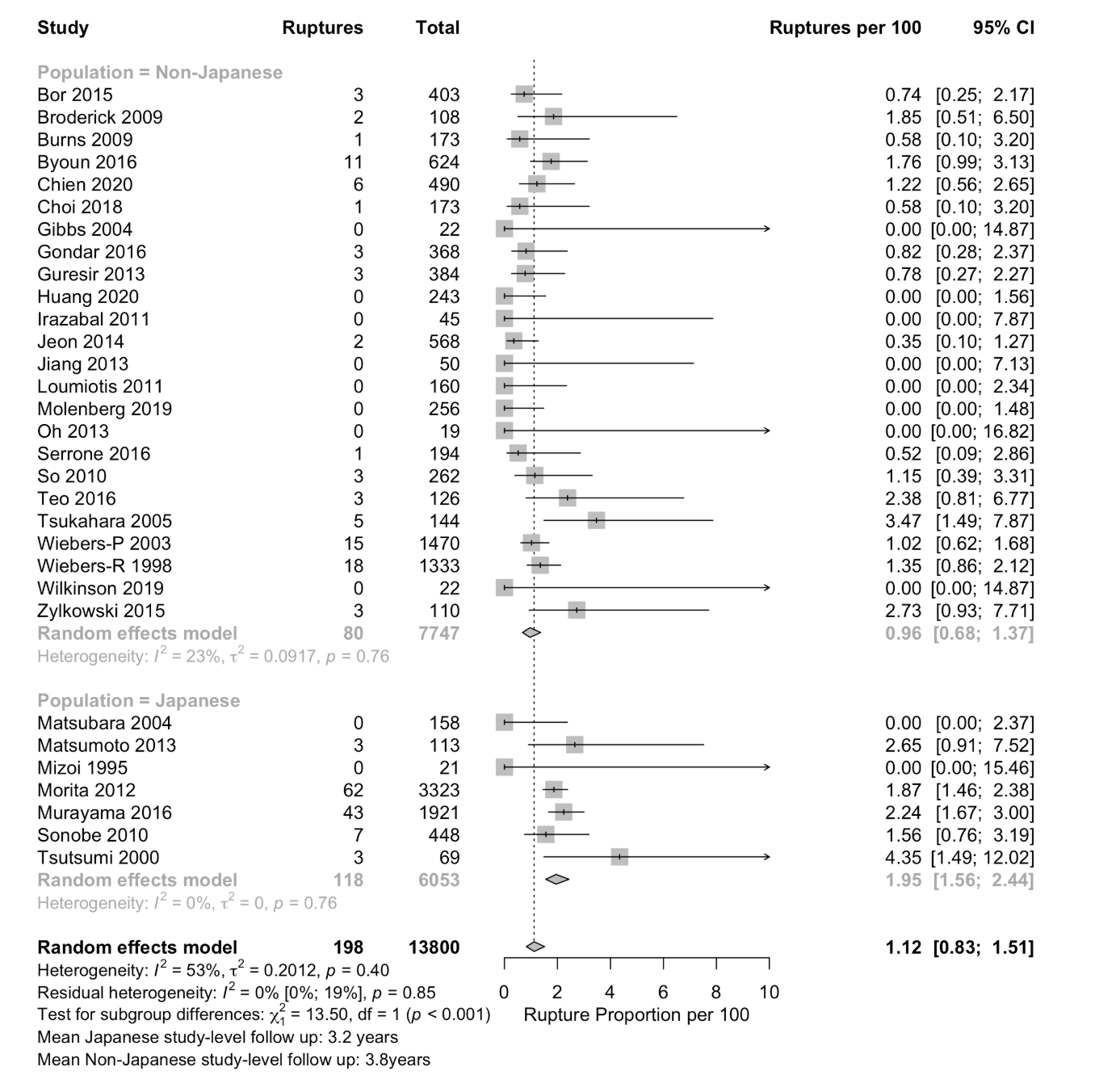


The test for subgroup differences (random-effects model) indicates that there is a statistically significant subgroup effect (*P*<.001), suggesting that a Japanese source population was associated with a higher proportion of rupture across the included studies. This covariate also explains some of the heterogeneity, with low heterogeneity remaining *I^2^* =0.0% 95% CI [0.0%; 19.4%]. The overall covariate distribution is balanced with the total number of rupture events and UIAs in each sub-group. However, this sub-group analysis is susceptible to aggregation bias and confounding bias from other study level characteristics - included Japanese samples were older (64.5-years vs 57.4-years, *P*=0.001) compared to non-Japanese samples.

## Supplementary Figure 6. Meta-regression of proportion of patients included with exposure to prior SAH as a continuous variable on proportion of rupture per 100 UIAs ≤10mm managed conservatively without repair.


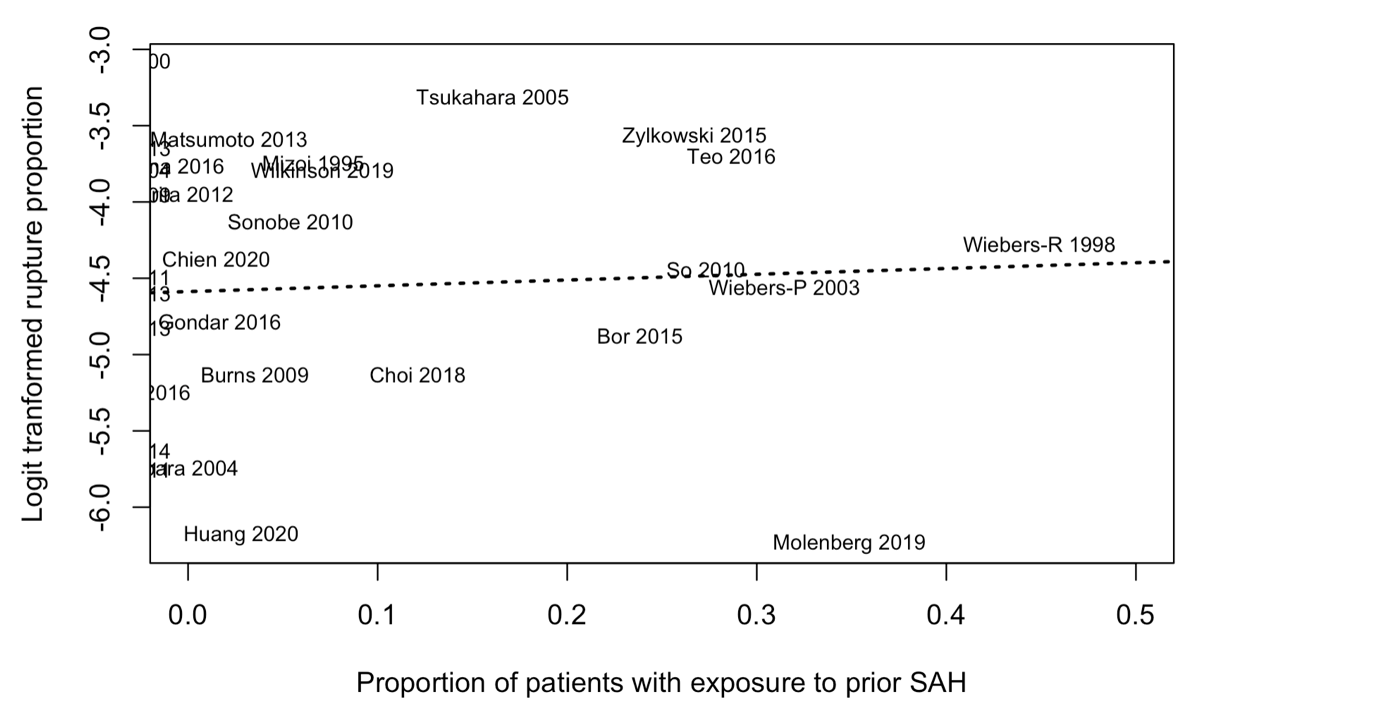


The random-effects meta-regression included participants from 30 studies with a mean study level proportion of exposure to prior subarachnoid haemorrhage of 11.9% (range 0 - 49.8%). The overall covariate distribution is not concerning with a total of 187 ruptures and 13176 aneurysms in studies included in this meta-regression analysis. The meta-regression result was not statistically significant (F(1,28)=0.15, P=0.70), suggesting that the proportion of patients with prior SAH included in the studies was not associated with the proportion of ruptureat the study level. There is moderate unexplained residual heterogeneity (*I^2^* = 54.1%), which is higher when all studies were included, indicating that this meta-regression analysis was not informative in identifying sources of heterogeneity in the meta-analysis result. In addition, this random-effects meta-regression analysis is susceptible to aggregation bias and confounding bias from other study level characteristics.

## Supplementary Figure 7. Meta-regression of proportion of patients included with ≤5mm aneurysms as a continuous variable of proportion of rupture per 100 UIAs ≤10mm managed conservatively without repair.


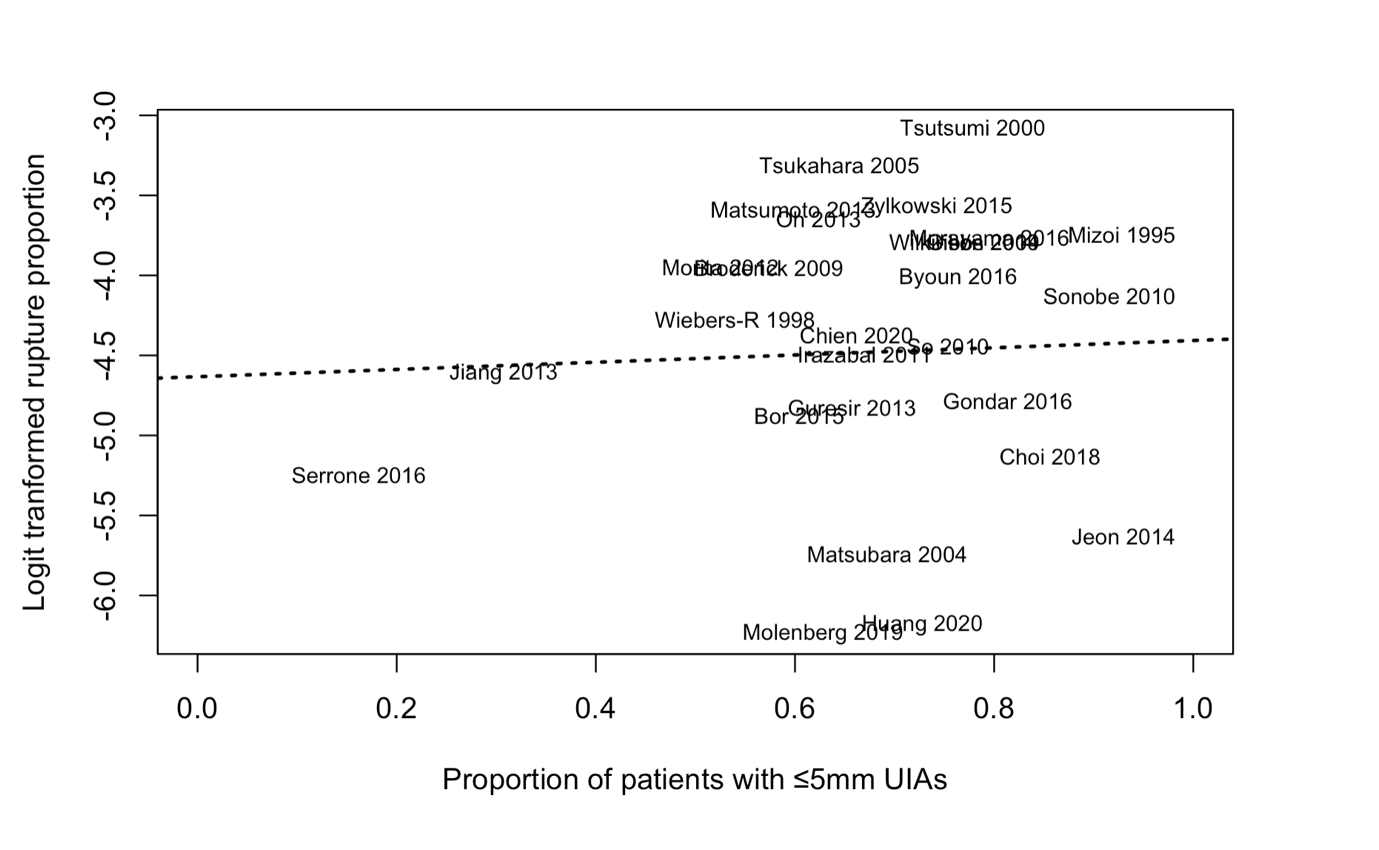


The random-effects meta-regression included participants from 27 studies with a mean study level proportion of 5mm aneurysms of 76.6% (range 24.7 - 100%). The overall covariate distribution is not concerning with a total of 179 ruptures and 11871 aneurysms in studies included in this meta-regression analysis. The meta-regression result was not statistically significant (F(1,25)=0.05, P=0.81), suggesting that the proportion of patients with ≤5mm UIAs included in the study was not associated with the proportion of rupture at the study level. There is moderate unexplained residual heterogeneity (*I^2^* = 50.3%), similar to that occurring when all studies were included, indicating that this meta-regression analysis was not informative in identifying sources of heterogeneity in the meta-analysis result. In addition, this random-effects meta-regression analysis is susceptible to aggregation bias and confounding bias from other study level characteristics.

## Supplementary Figure 8. Meta-regression of proportion of patients included with multiple aneurysms as a continuous variable on proportion of rupture per 100 UIAs ≤10mm managed conservatively without repair.


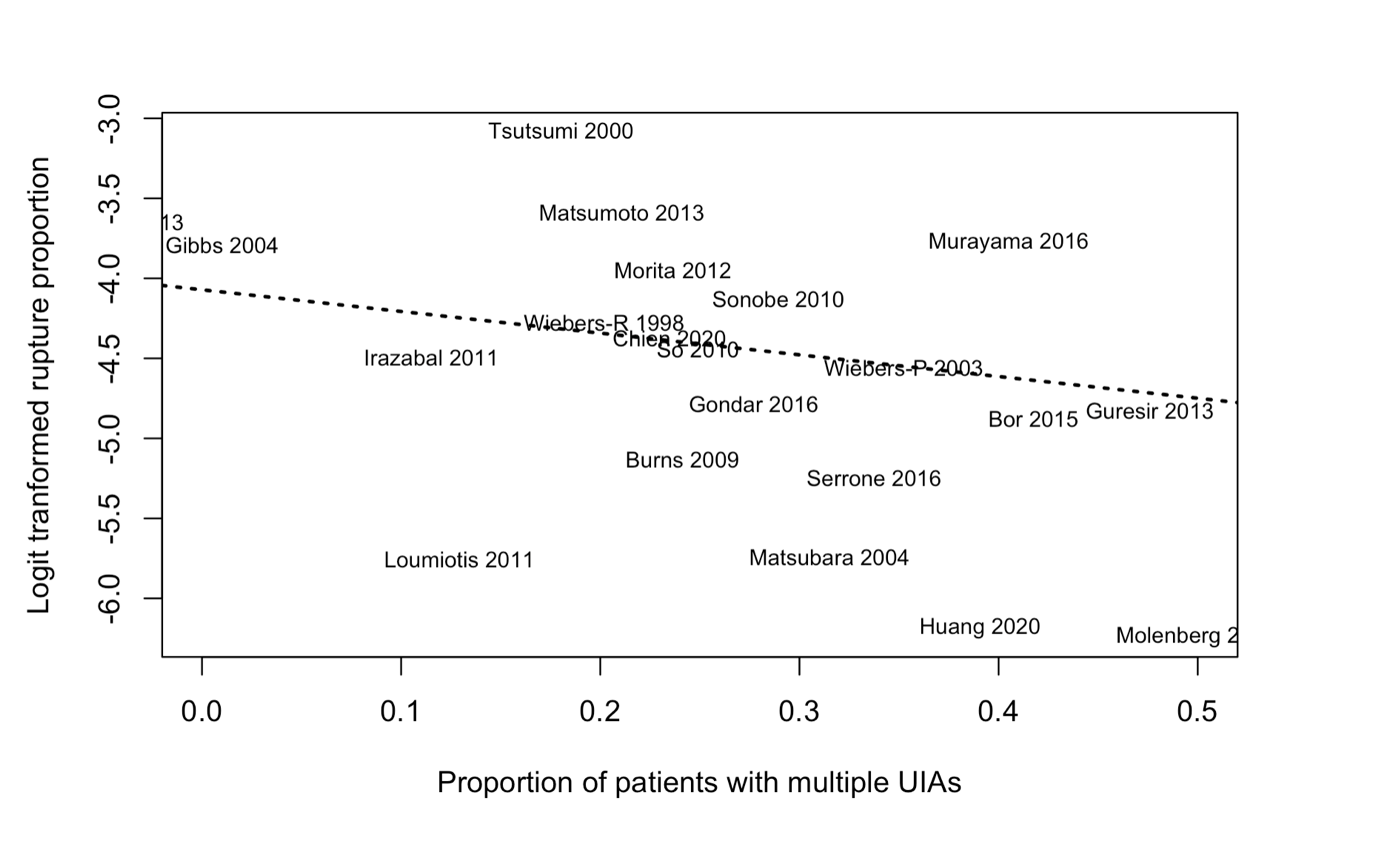


The random-effects meta-regression included participants from 21 studies with a mean study level proportion of aneurysm multiplicity of 30.6% (range 0 - 54.9%). The overall covariate distribution is not concerning with a total of 171 ruptures and 11854 aneurysms in studies included in this meta-regression analysis. The meta-regression result was not statistically significant (F(1,19)=0.80, *P*= 0.38), suggesting that the proportion of patients with multiple aneurysms included in the study was not associated with the proportion of rupture at the study level. However, there is moderate unexplained residual heterogeneity (*I^2^* = 50.8%), similar to that occurring when all studies were included, indicating that this meta-regression analysis was not informative in identifying sources of heterogeneity in the meta-analysis result. In addition, this random-effects meta-regression analysis is susceptible to aggregation bias and confounding bias from other study level characteristics.

## Supplementary Figure 9. Meta-regression of proportion of patients included with irregular shaped aneurysms as a continuous variable on proportion of rupture per 100 UIAs ≤10mm managed conservatively without repair.


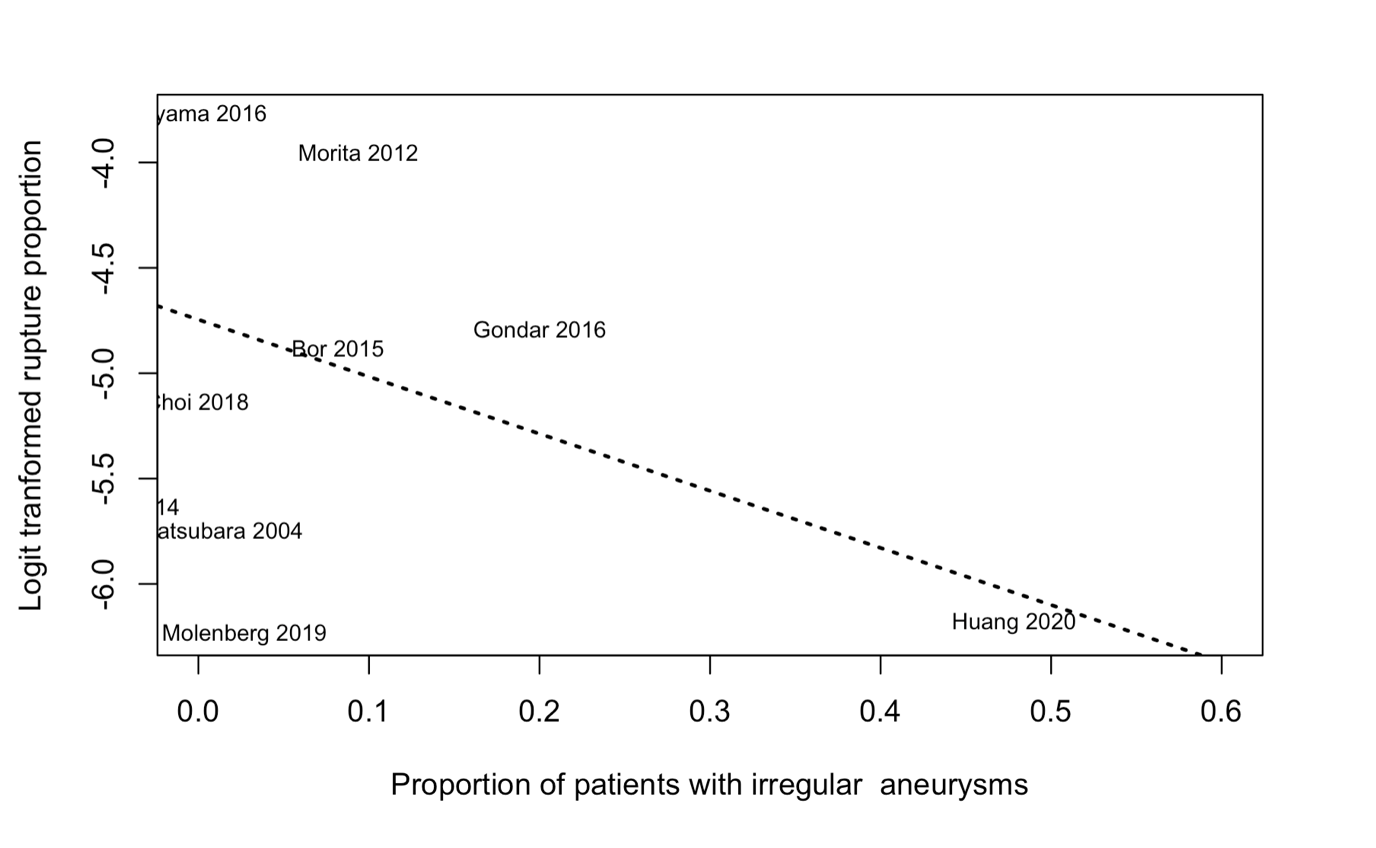


The random-effects meta-regression included participants from 9 studies with a mean study level proportion of irregular aneurysms of 14.3% (range 0 - 52.5%). The overall covariate distribution is not concerning with a total of 114 ruptures and 7 413 aneurysms in studies included in this meta-regression analysis. The meta-regression result was not significant F(1,7)=0.67, p-val = 0.44 suggesting that the proportion of patients with irregular aneurysms included in the studies was not associated with the rupture proportion at the study level. There is high unexplained residual heterogeneity (I^2^ = 80.5%), which is higher than across all included studies, indicating that this meta-regression analysis was not informative in identifying sources of heterogeneity in the meta-analysis result. In addition, this random-effects meta-regression analysis is susceptible to aggregation bias and confounding bias from other study level characteristics.

## Supplementary Figure 10. Meta-regression of proportion of patients included with posterior circulation aneurysms as a continuous variable on proportion of rupture per 100 UIAs ≤10mm managed conservatively without repair.


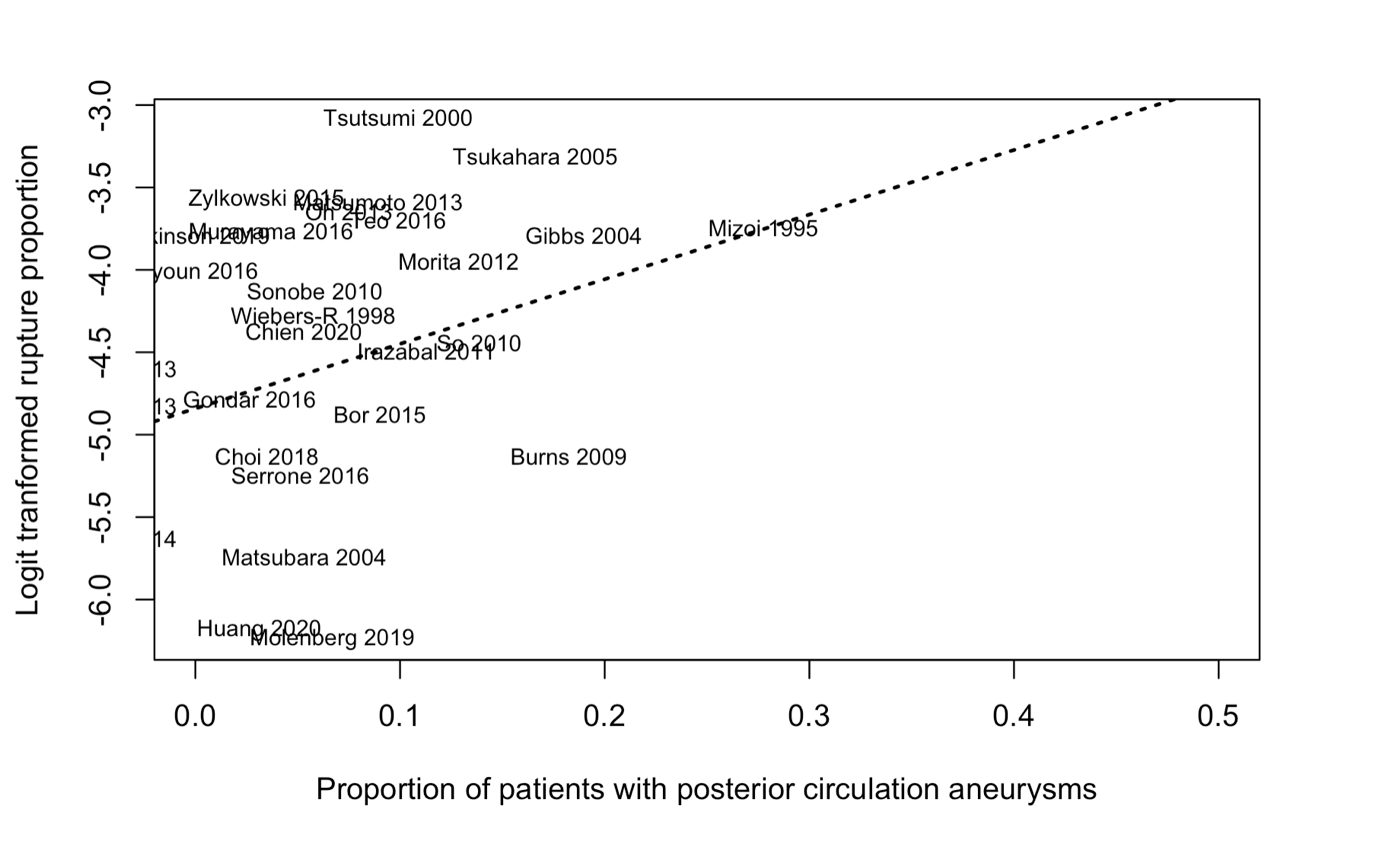


The random-effects meta-regression included participants from 28 studies with a mean study level proportion of posterior circulation aneurysms of 11.4% (range 0 - 31.3%). The overall covariate distribution is not concerning with a total of 181 ruptures and 12 602 aneurysms in studies included in this meta-regression analysis. The meta-regression result was not significant F(1, 26) = 2.97, p-val = 0.10 suggesting that the proportion of patients with posterior circulation aneurysms included in the studies was not associated with the rupture proportion at the study level. There is moderate unexplained residual heterogeneity (I^2^ = 42.4%), which is similar to across all included studies, indicating that this meta-regression analysis was not informative in identifying sources of heterogeneity in the meta-analysis result. In addition, this random-effects meta-regression analysis is susceptible to aggregation bias and confounding bias from other study level characteristics.

## Supplementary Figure 11. Meta-regression of proportion of patients included with anterior circulation aneurysms as a continuous variable on proportion of rupture per 100 UIAs ≤10mm managed conservatively without repair.


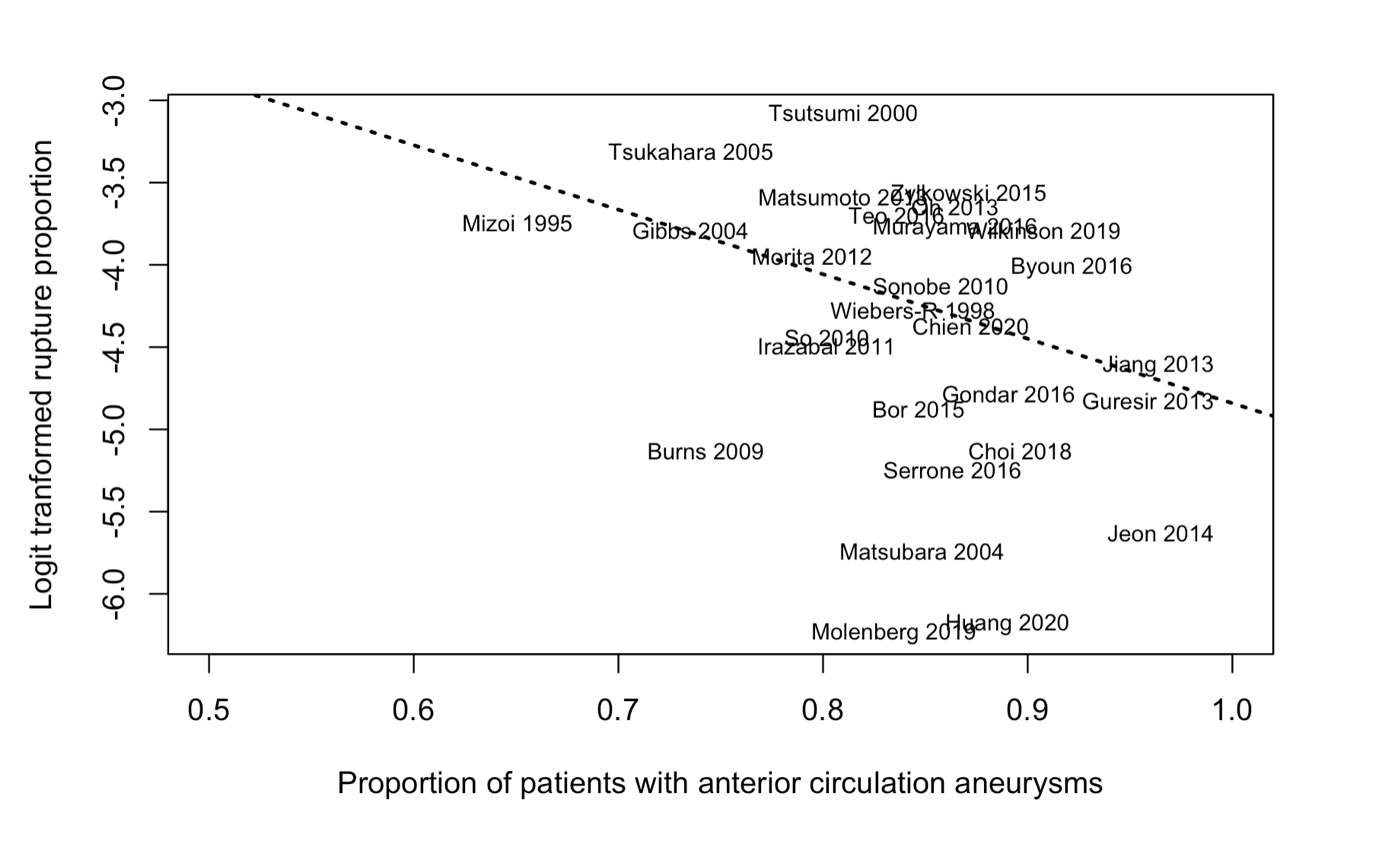


The random-effects meta-regression included participants from 28 studies with a mean study level proportion of anterior circulation aneurysms of 88.6% (range 68.7 - 100%). The overall covariate distribution is not concerning with a total of 181 ruptures and 12 602 aneurysms in studies included in this meta-regression analysis. The meta-regression result was not significant F(1, 26) = 2.97, p-val = 0.10 suggesting that the proportion of patients with anterior circulation aneurysms included in the studies was not associated with the rupture proportion at the study level. There is moderate unexplained residual heterogeneity (I^2^ = 42.5%), which is similar to across all included studies, indicating that this meta-regression analysis was not informative in identifying sources of heterogeneity in the meta-analysis result. In addition, this random-effects meta-regression analysis is susceptible to aggregation bias and confounding bias from other study level characteristics.

## Supplementary Figure 12. Meta-regression of proportion of patients included with anterior communicating artery or anterior cerebral aneurysms as a continuous variable on proportion of rupture per 100 UIAs ≤10mm managed conservatively without repair.


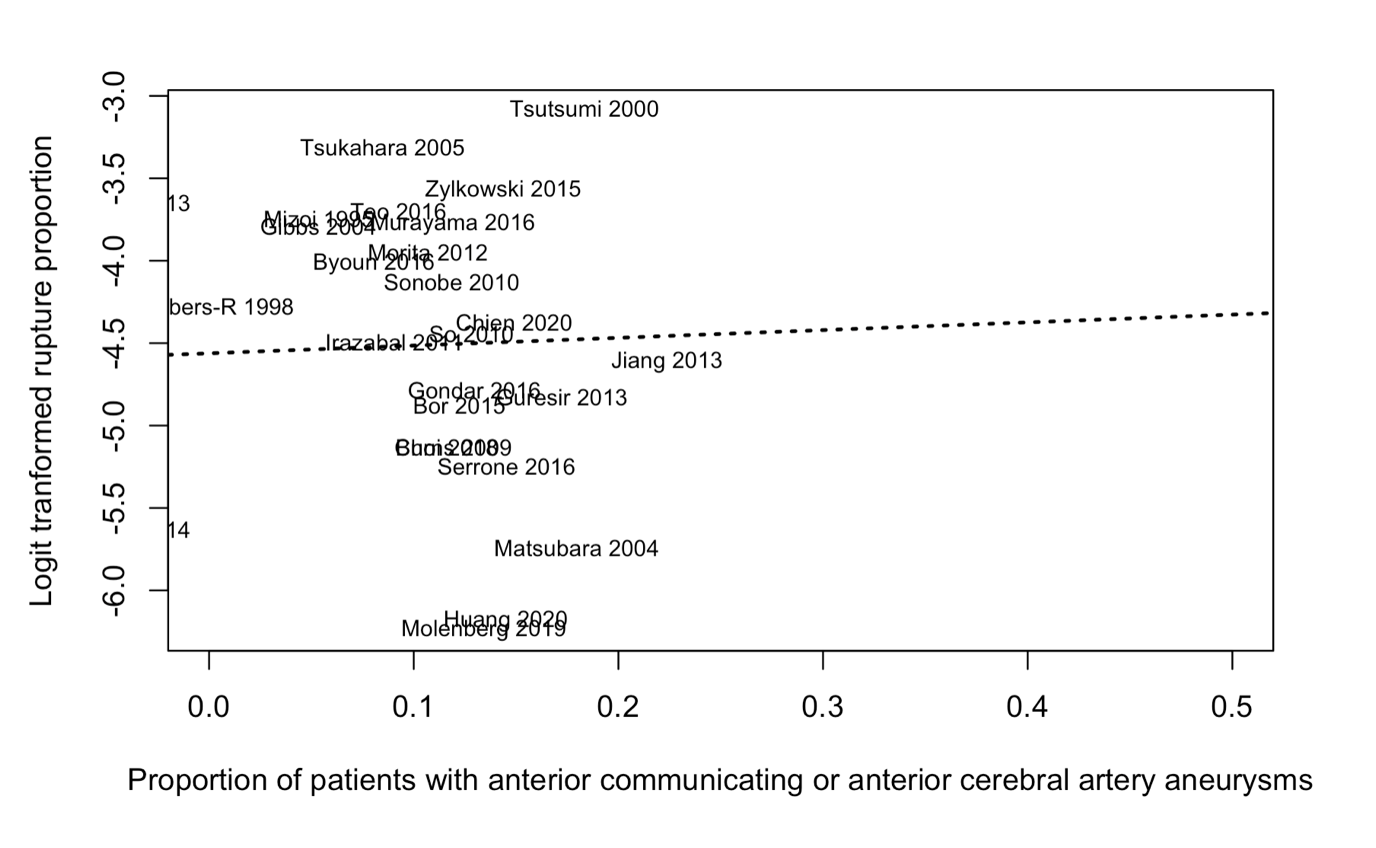


The random-effects meta-regression included participants from 26 studies with a mean study level proportion of anterior communicating or anterior cerebral artery aneurysms of 14.9% (range 0 - 26%). The overall covariate distribution is not concerning with a total of 178 ruptures and 11 927 aneurysms in studies included in this meta-regression analysis. The meta-regression result was not significant F(1, 24)= 0.03, p-val = 0.87 suggesting that the proportion of patients with anterior communicating or anterior cerebral artery aneurysms included in the studies was not associated with the rupture proportion at the study level. There is moderate unexplained residual heterogeneity (I^2^ = 56.4%), which is similar to across all included studies, indicating that this meta-regression analysis was not informative in identifying sources of heterogeneity in the meta-analysis result. In addition, this random-effects meta-regression analysis is susceptible to aggregation bias and confounding bias from other study level characteristics.

## Supplementary Figure 13. Meta-regression of age as a continuous variable on proportion of rupture per 100 UIAs ≤10mm managed conservatively without repair.


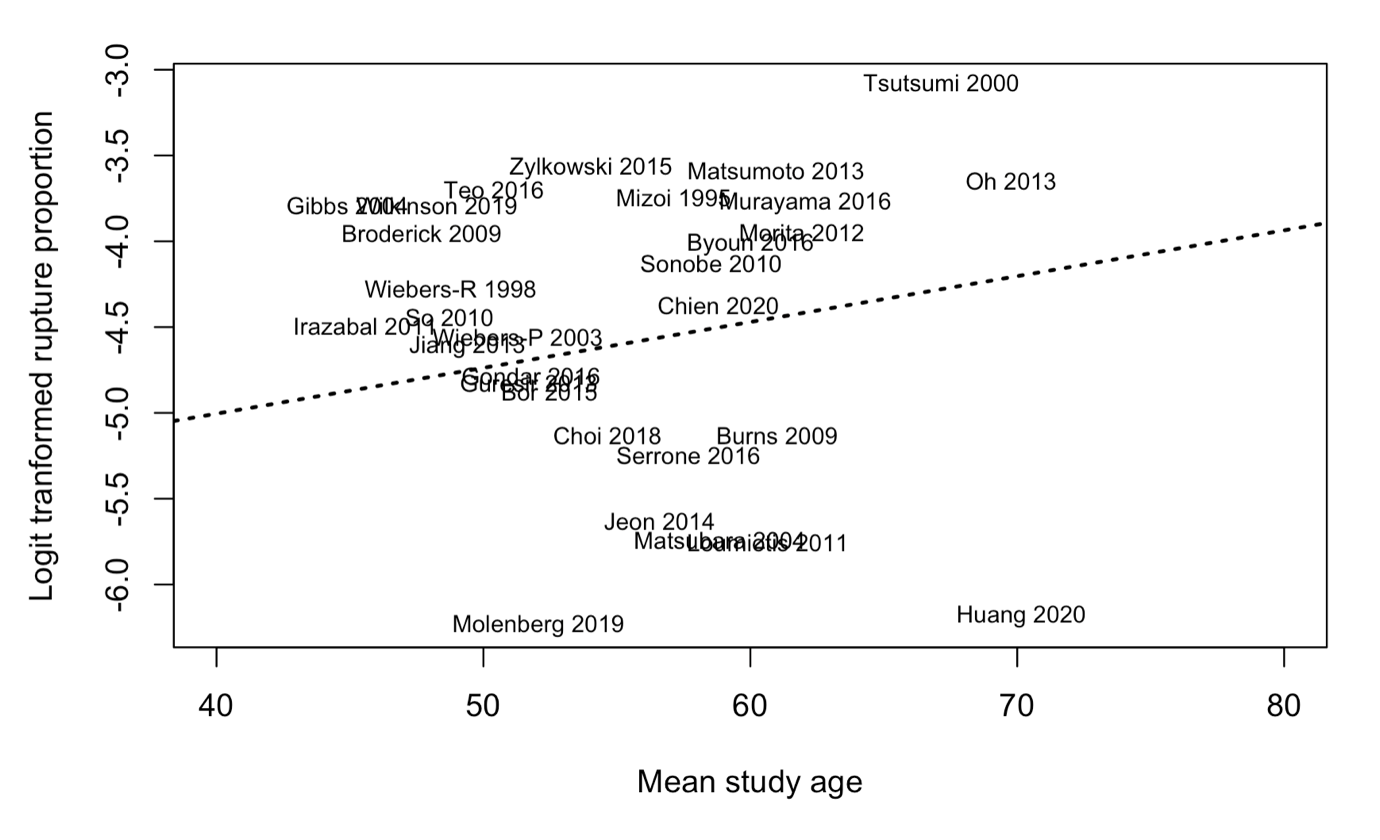


This random effect meta-regression analysis included participants from 30 studies with a mean study level patient age of 59.1 +/- 6.8 years (range 47.9-73.3 years). There was a total of 193 ruptures and 13656 aneurysms in studies included in this meta-regression analysis. The meta-regression result was not statistically significant (F(1,28)=1.41, *P*=0.25) suggesting that increasing study-level age was not associated with the proportion of rupture at the study level. There is moderate unexplained residual heterogeneity (*I^2^* = 38.2%), indicating that this meta-regression analysis was not informative in identifying sources of heterogeneity in the meta-analysis result. However, this analysis is susceptible to aggregate data bias and confounding bias from other study level characteristics.

## Supplementary Figure 14. Sub-group analysis of study type per 100 UIAs ≤10mm managed conservatively without repair categorised as prospective or retrospective.


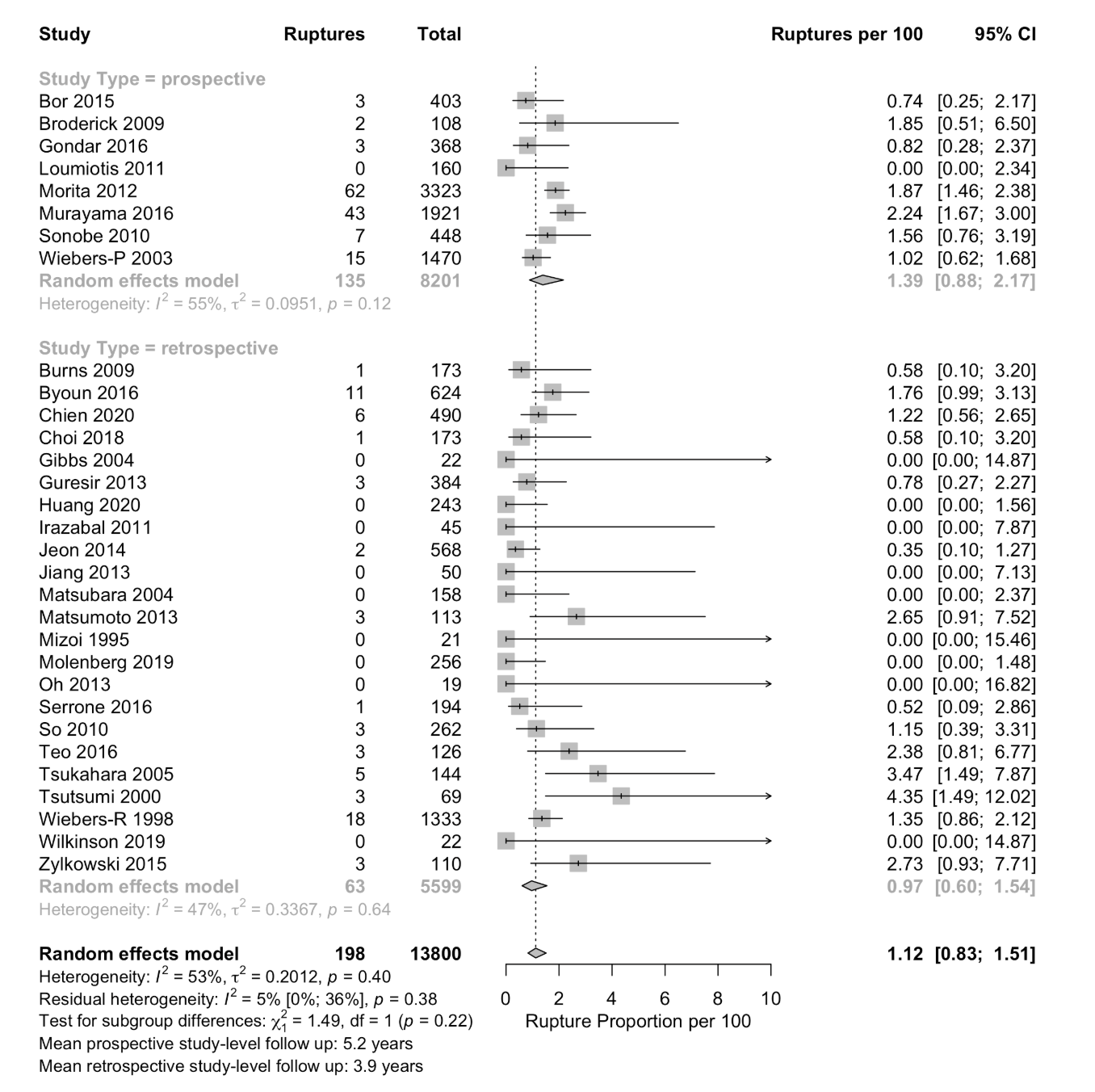


The test for subgroup differences (random-effects model) indicates that there is no statistically significant subgroup effect (*P*= 0.22), suggesting that there was no difference in proportion of rupture between prospective and retrospective studies. Although retrospective studies may be at greater risk of incomplete case follow up due to the case-fatality risk prior to hospital presentation, this sub-group analysis demonstrates no clinically relevant difference in estimated rupture proportion. This covariate does explain some of the residual heterogeneity, with low heterogeneity remaining *I^2^* =5.3% 95% CI [0.0%; 35.6%]. There is also some concern regarding the overall covariate distribution given majority of the rupture events are in the prospective cohort, with most rupture events occurring in 2 studies with a Japanese source population. In addition, there may be additional unknown confounders also influencing the results of the subgroup analysis.

## Supplementary Figure 15. Meta-regression of follow up time as a continuous variable on proportion of rupture per 100 UIAs ≤10mm managed conservatively without repair.


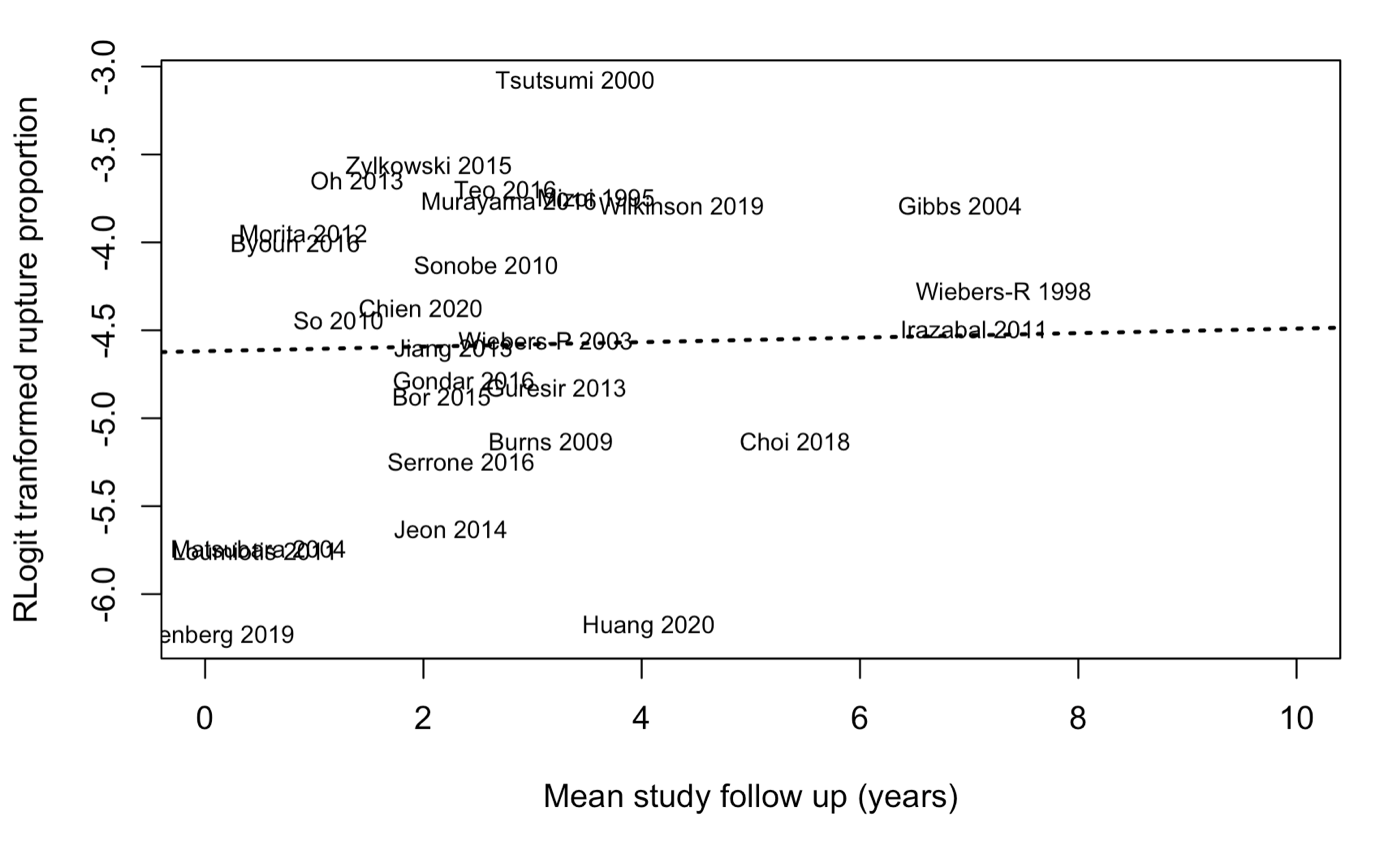


The random-effects meta-regression included participants from 28 studies with a mean study level follow up time of 3.7 years (range 1.0 - 8.3 years). There were a total of 188 ruptures and 13435 aneurysms in studies included in this meta-regression analysis. The meta-regression result was not statistically significant (F(1,26)=0.03, *P*=0.88), suggesting that mean study level follow up time across the studies was not associated with the rupture proportion. There is moderate unexplained residual heterogeneity (*I^2^* = 52.0%), similar to that occurring when all studies were included, indicating that this meta-regression analysis was not informative in identifying sources of heterogeneity in the meta-analysis result. In addition, this analysis is susceptible to aggregate data bias and confounding bias from other study level characteristics.

## Supplementary Figure 16. Sensitivity analysis using leave-one-out method ordered by impact on the pooled proportion of rupture with residual *I^2^* indicated.


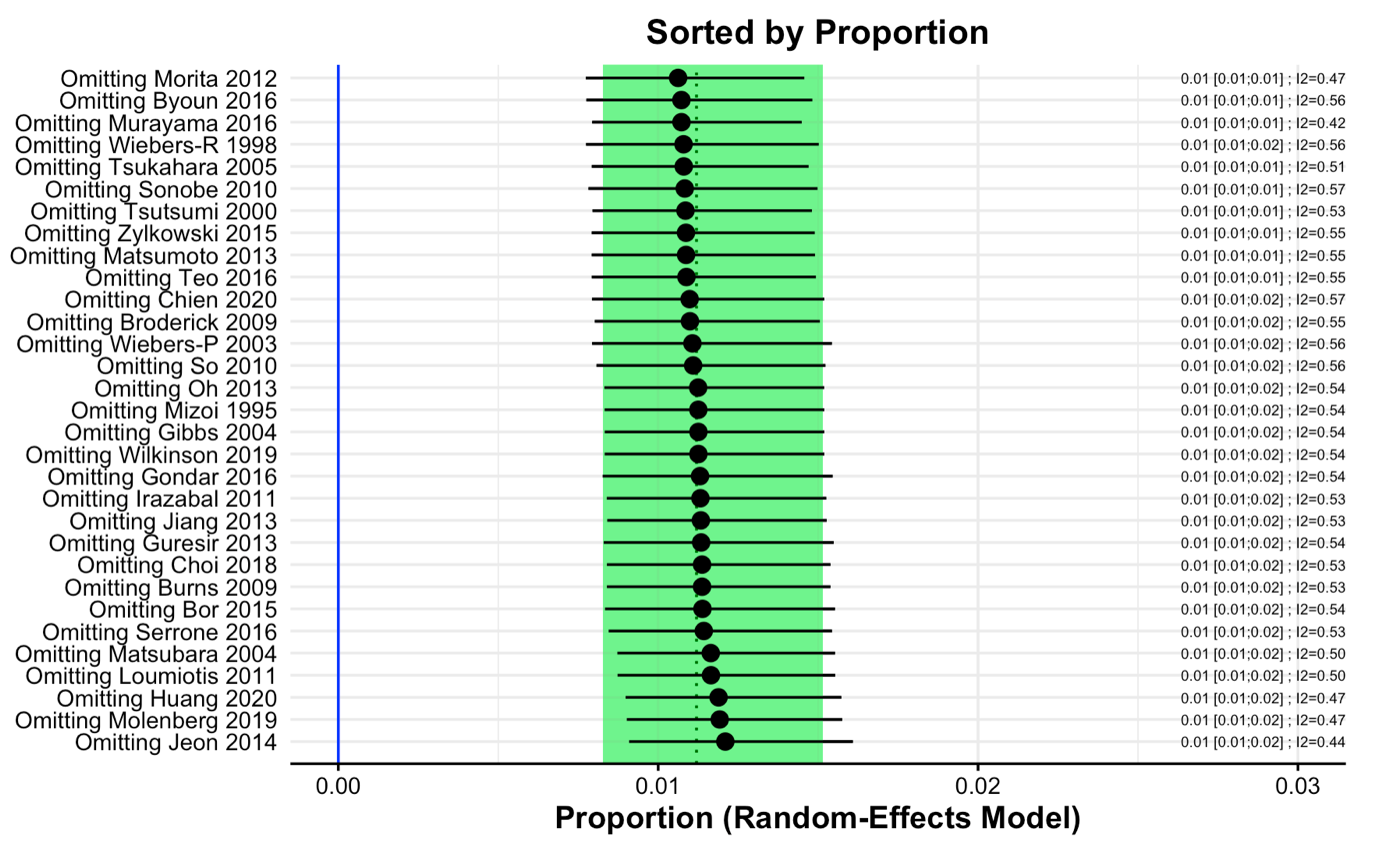


There is no clinically relevant effect in the pooled proportion of ruptures.

## Supplementary Figure 17. Sensitivity analysis using ≤7mm UIA size threshold.


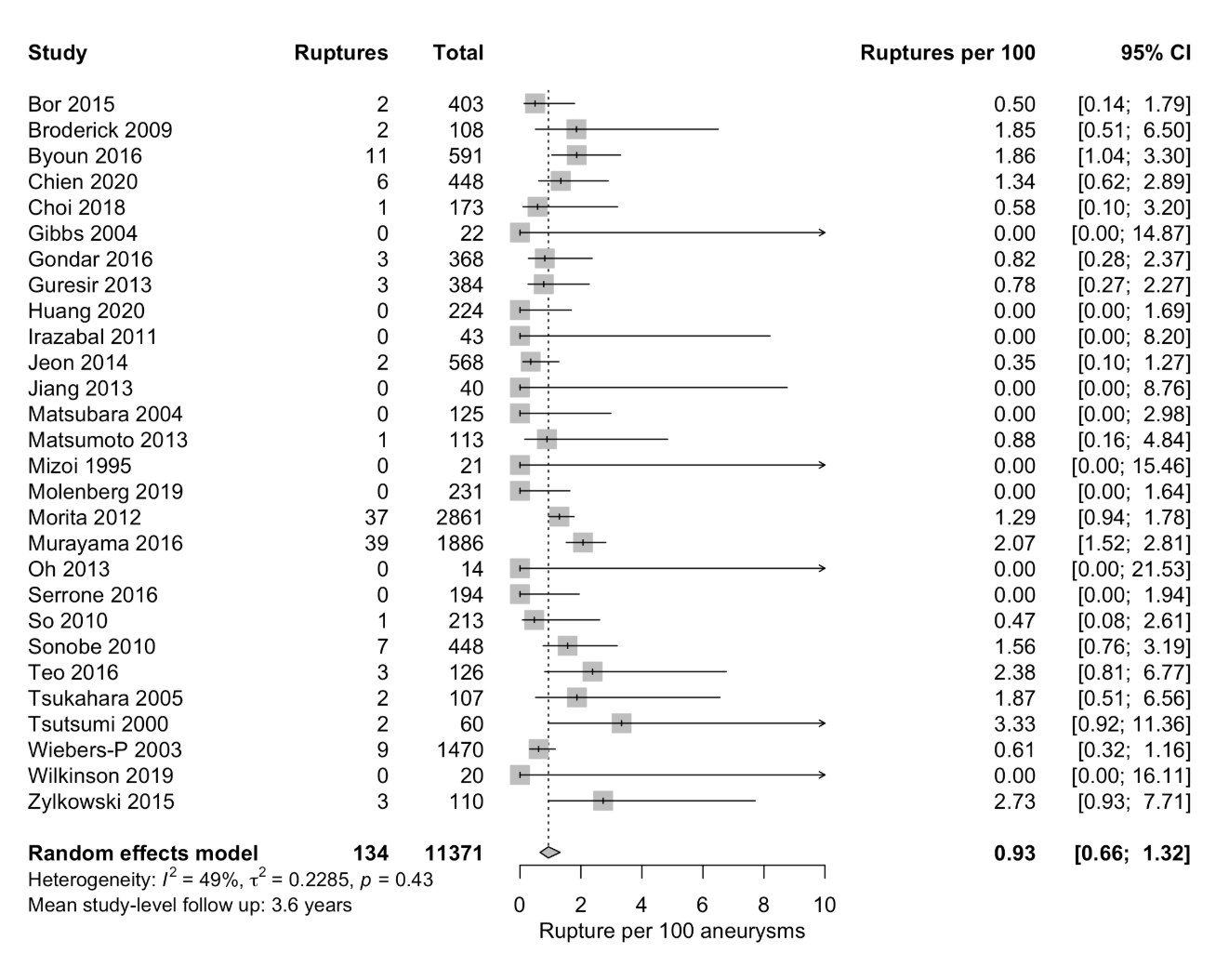


There is no clinically relevant effect in the pooled proportion of ruptures.

## Supplementary Figure 18. Sensitivity analysis using ≤5mm UIA size threshold.


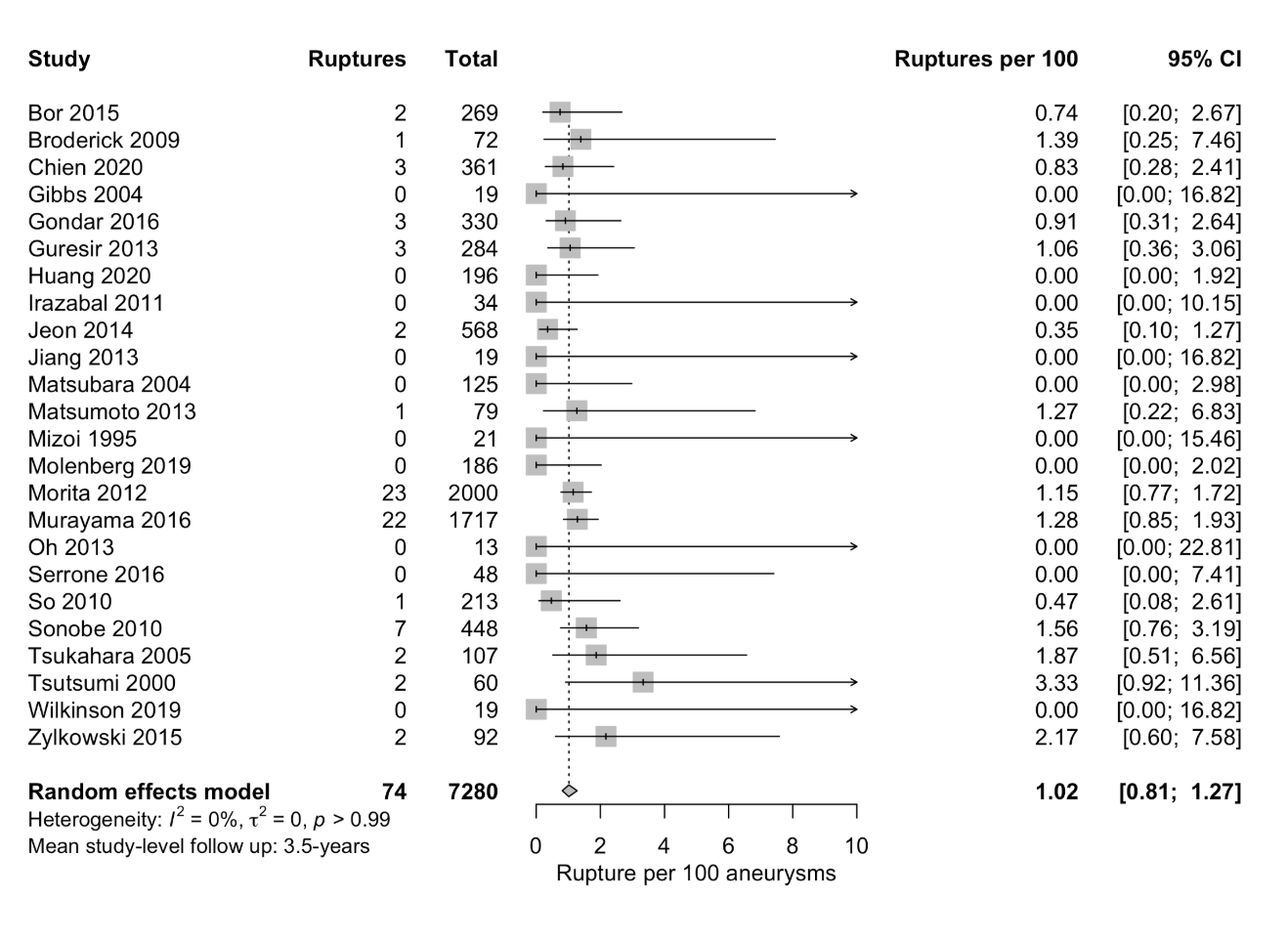


There is no clinically relevant effect in the pooled proportion of ruptures.

## Supplementary Figure 19. Sensitivity analysis using ≤3mm UIA size threshold.


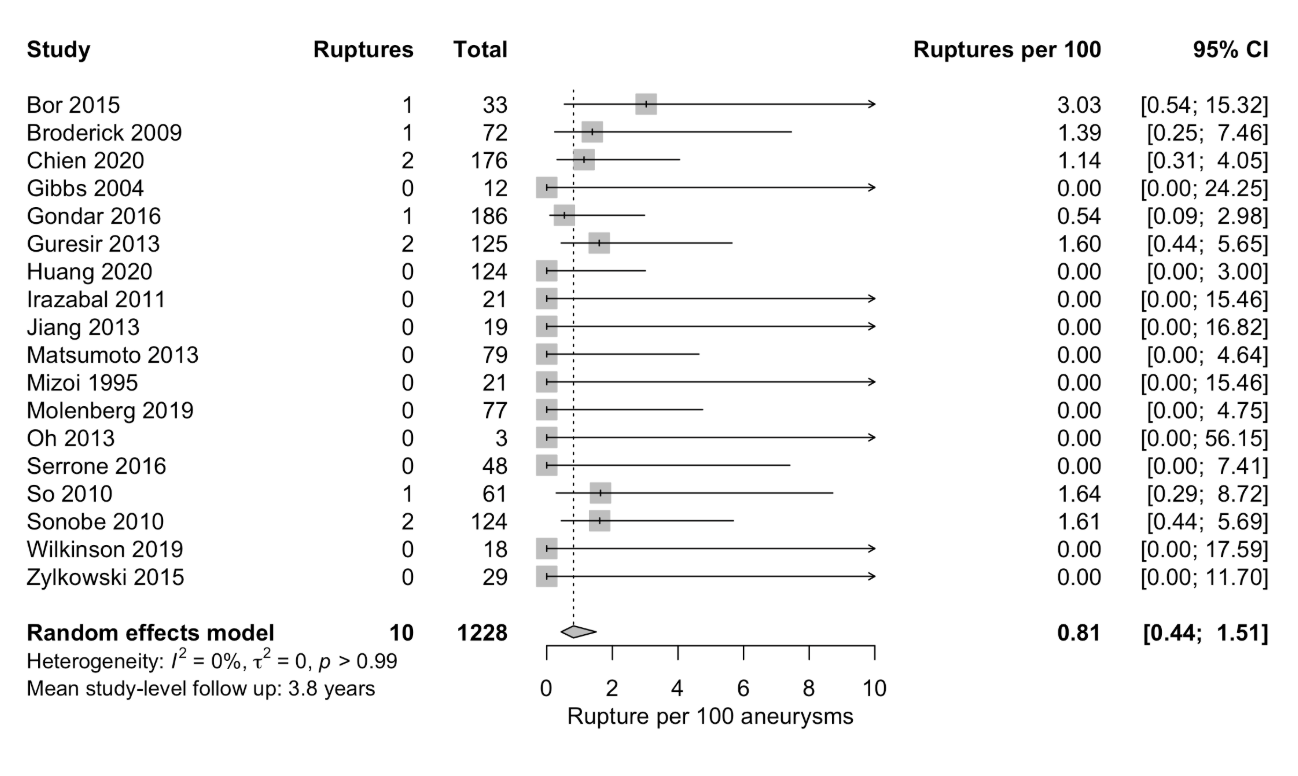


There is no clinically relevant effect in the pooled proportion of ruptures.

## Supplementary Figure 20. Sensitivity analysis including outlier study.


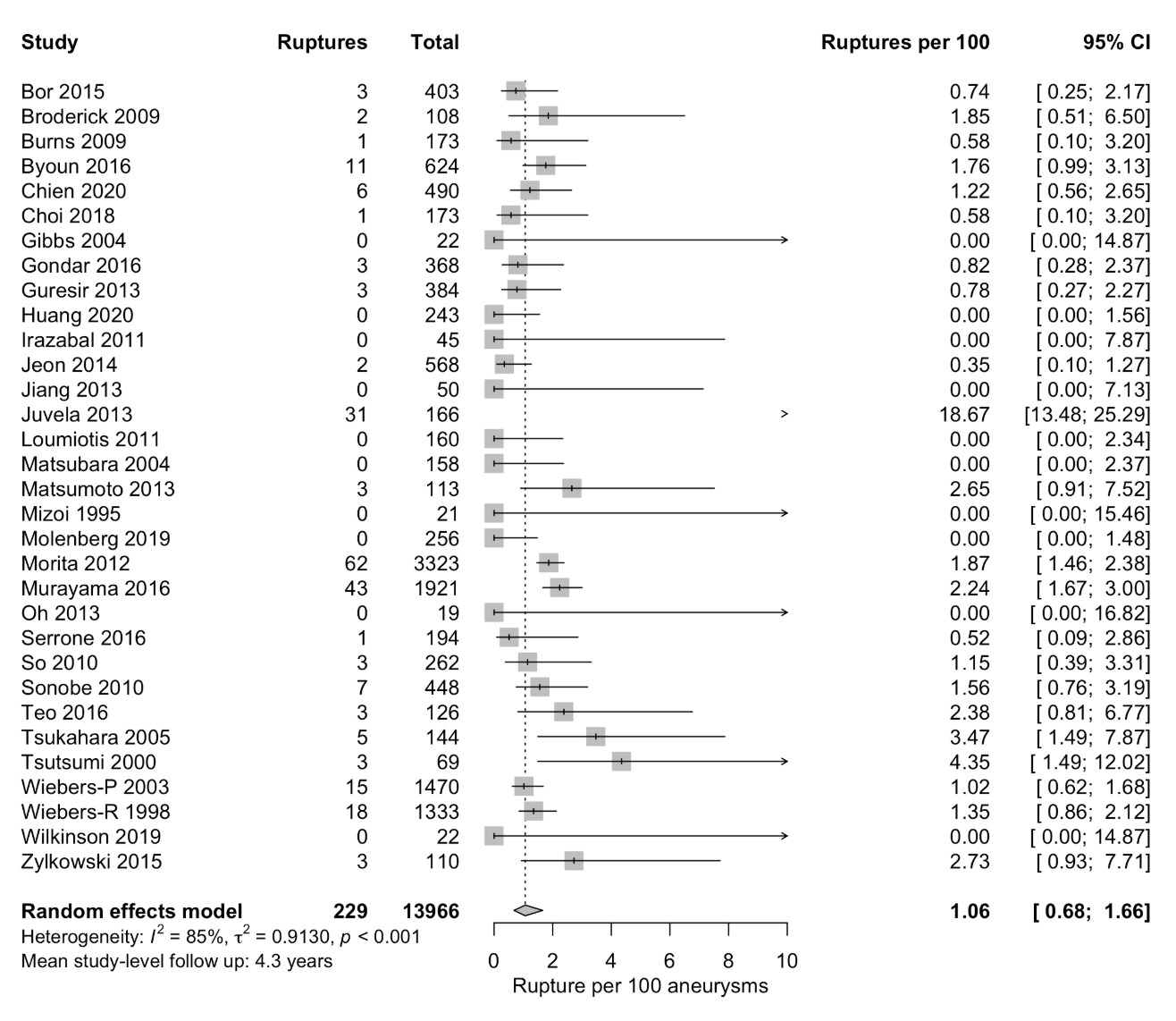


There is no clinically relevant effect in the pooled proportion of ruptures.

## Supplementary Figure 21. Sensitivity analysis limited to good standard studies.


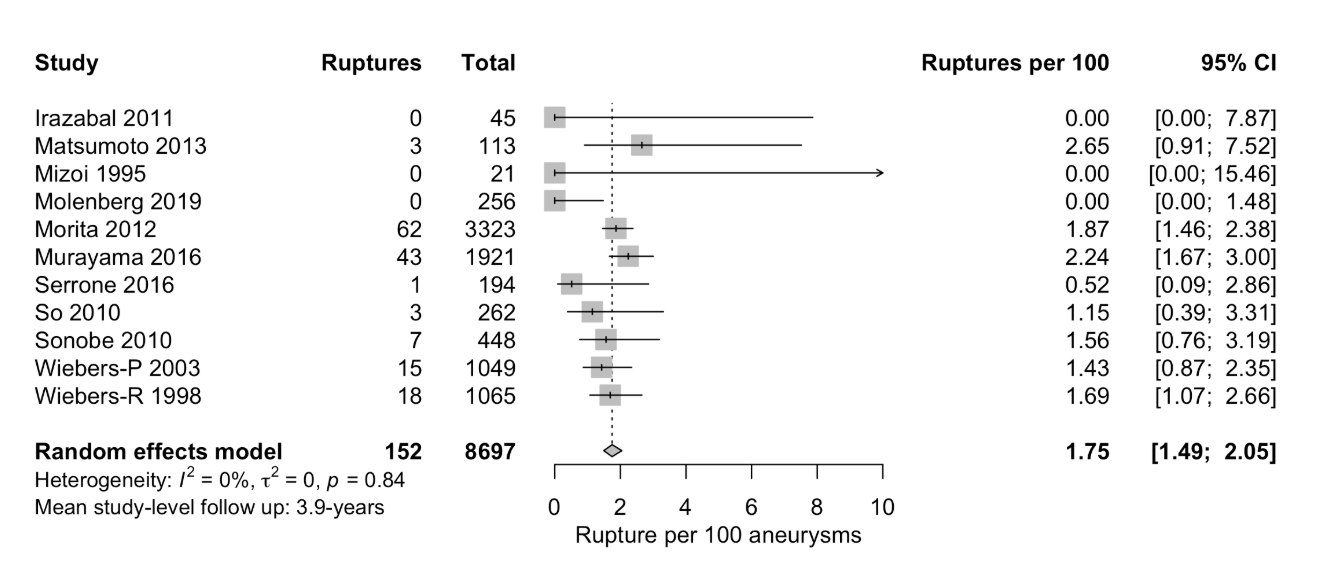


There is no clinically relevant effect in the pooled proportion of ruptures.

Good quality as per the Agency for Healthcare Research and Quality(13) standard: 3 or 4 stars in selection domain AND 1 or 2 stars in comparability domain AND 2 or 3 stars in outcome/exposure domain.

## Supplementary Figure 22. Funnel plot to examine small-study effects included in meta-analysis.


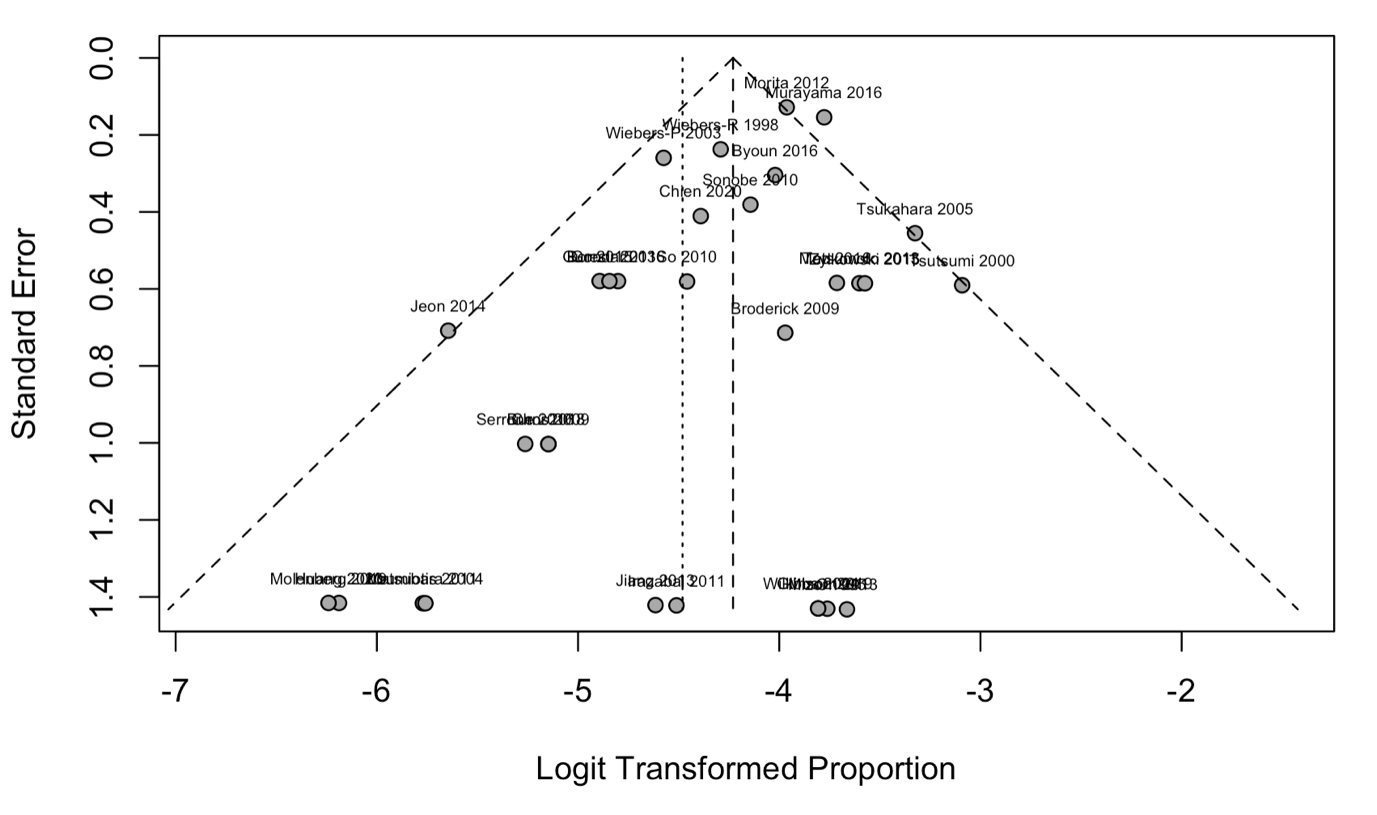


Egger’s test *P*=0.01

Multiple possible sources of asymmetry other than random chance were identifiable. These include non-reporting bias, selective reporting bias, small-study effects, and residual heterogeneity.

## Supplementary Figure 23. Sensitivity analysis to consider small-study effects.


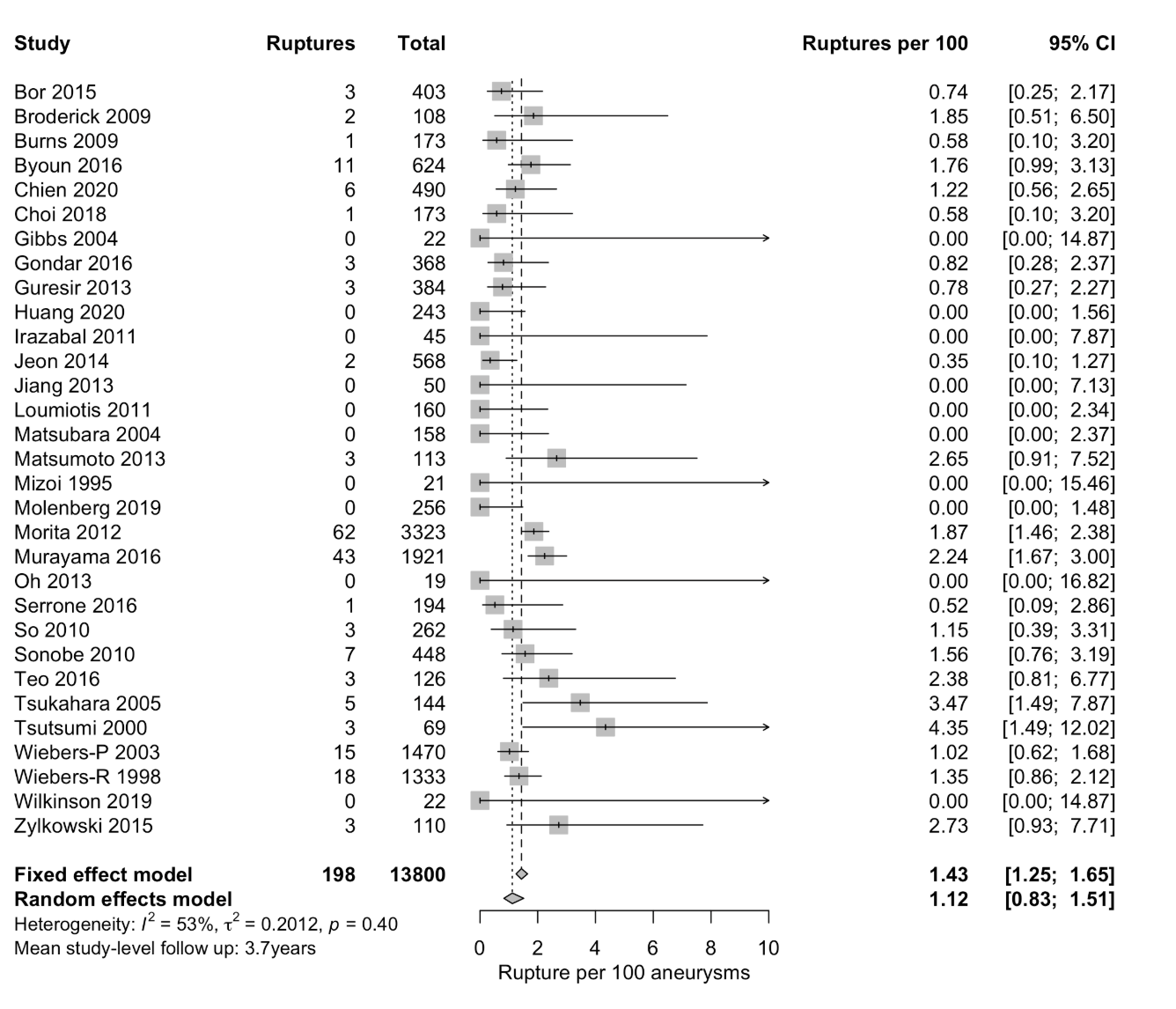


Examination of the random-effects and fixed-effects estimates are similar, with no clinically relevant impact in the estimate of the pooled proportion of rupture. Thus, small-study effects have no clinically relevant effect on the pooled rupture proportion.

**Supplementary References**

1. Schwarzer G, Chemaitelly H, Abu-Raddad LJ, Rucker G. Seriously misleading results using inverse of Freeman-Tukey double arcsine transformation in meta-analysis of single proportions. *Res Synth Methods* (2019) 10(3):476-83. Epub 2019/04/05. doi: 10.1002/jrsm.1348. PubMed PMID: 30945438; PubMed Central PMCID: PMCPMC6767151.

2. Stijnen T, Hamza TH, Ozdemir P. Random-effects meta-analysis of event outcome in the framework of the generalized linear mixed model with applications in sparse data. *Stat Med* (2010) 29(29):3046-67. Epub 2010/09/10. doi: 10.1002/sim.4040. PubMed PMID: 20827667.

3. Hamza TH, van Houwelingen HC, Stijnen T. The binomial distribution of meta-analysis was preferred to model within-study variability. *J Clin Epidemiol* (2008) 61(1):41-51. Epub 2007/12/18. doi: 10.1016/j.jclinepi.2007.03.016. PubMed PMID: 18083461.

4. Hartung J, Knapp G. A refined method for the meta-analysis of controlled clinical trials with binary outcome. *Stat Med* (2001) 20(24):3875-89. Epub 2002/01/10. doi: 10.1002/sim.1009. PubMed PMID: 11782040.

5. Deeks JJ HJ, Altman DG. Chapter 10: Analysing data and undertaking meta-analyses. In: Higgins JPT TJ, Chandler J, Cumpston M, Li T, Page MJ, Welch VA, editor. *Cochrane Handbook for Systematic Reviews of Interventions version 60 (updated July 2019)*. Cochrane (2019).

6. Brown L, Cai TT, Das Gupta A. Interval Estimation for a Binomial Proportion. *Stat Sci* (2001) 16(2):101-33.

7. Newcombe RG. Two-sided confidence intervals for the single proportion: comparison of seven methods. *Stat Med* (1998) 17(8):857-72. Epub 1998/05/22. doi: 10.1002/(sici)1097-0258(19980430)17:8<857::aid-sim777>3.0.co;2-e. PubMed PMID: 9595616.

8. Knapp G, Hartung J. Improved tests for a random-effects meta-regression with a single covariate. *Stat Med* (2003) 22(17):2693-710. Epub 2003/08/27. doi: 10.1002/sim.1482. PubMed PMID: 12939780.

9. Higgins JP, Thompson SG. Controlling the risk of spurious findings from meta-regression. *Stat Med* (2004) 23(11):1663-82. Epub 2004/05/26. doi: 10.1002/sim.1752. PubMed PMID: 15160401.

10. Viechtbauer W, Lopez-Lopez JA, Sanchez-Meca J, Marin-Martinez F. A comparison of procedures to test for moderators in mixed-effects meta-regression models. *Psychol Methods* (2015) 20(3):360-74. Epub 2014/08/12. doi: 10.1037/met0000023. PubMed PMID: 25110905.

11. Thompson SG, Higgins JP. How should meta-regression analyses be undertaken and interpreted? *Stat Med* (2002) 21(11):1559-73. Epub 2002/07/12. doi: 10.1002/sim.1187. PubMed PMID: 12111920.

12. Juvela S, Poussa K, Lehto H, Porras M. Natural history of unruptured intracranial aneurysms: a long-term follow-up study. *Stroke* (2013) 44(9):2414-21. doi: <https://dx.doi.org/10.1161/STROKEAHA.113.001838>. PubMed PMID: 23868274.

13. Viswanathan M, Ansari MT, Berkman ND, Chang S, Hartling L, McPheeters M, et al. Assessing the Risk of Bias of Individual Studies in Systematic Reviews of Health Care Interventions. *Methods Guide for Effectiveness and Comparative Effectiveness Reviews*. AHRQ Methods for Effective Health Care. Rockville (MD)(2012).
